# Adaptive Evolution in Zinc Finger Transcription Factors

**DOI:** 10.1371/journal.pgen.1000325

**Published:** 2009-01-02

**Authors:** Ryan O. Emerson, James H. Thomas

**Affiliations:** Department of Genome Sciences, University of Washington, Seattle, Washington, United States of America; University of Oxford, United Kingdom

## Abstract

The majority of human genes are conserved among mammals, but some gene families have undergone extensive expansion in particular lineages. Here, we present an evolutionary analysis of one such gene family, the poly–zinc-finger (poly-ZF) genes. The human genome encodes approximately 700 members of the poly-ZF family of putative transcriptional repressors, many of which have associated KRAB, SCAN, or BTB domains. Analysis of the gene family across the tree of life indicates that the gene family arose from a small ancestral group of eukaryotic zinc-finger transcription factors through many repeated gene duplications accompanied by functional divergence. The ancestral gene family has probably expanded independently in several lineages, including mammals and some fishes. Investigation of adaptive evolution among recent paralogs using d_N_/d_S_ analysis indicates that a major component of the selective pressure acting on these genes has been positive selection to change their DNA-binding specificity. These results suggest that the poly-ZF genes are a major source of new transcriptional repression activity in humans and other primates.

## Author Summary

Gene families, arising by the repeated duplication and diversification of existing genes, are a pervasive feature of the genomes of higher organisms. In this study, we analyze the evolutionary history of one of the largest gene families in humans, the poly–zinc-finger genes. Each poly–zinc-finger gene is thought to act by regulating the expression levels of one or more other genes, but the ultimate function and purpose of most poly–zinc-finger genes is unknown. We have found that the poly–zinc-finger gene family has been growing rapidly in many lineages including the human lineage, and that evolution has favored the creation of new poly–zinc-finger genes that have different DNA targets than the genes from which they were derived. These results suggest that the emergence of new and different poly–zinc-finger genes has probably been important in the evolution of humans and many other animal species.

## Introduction

Coordinated evolution of complex characters can occur by altering regulatory genes that control large suites of effector genes [1]. A small number of changes in these regulatory genes can result in conspicuous and harmonious changes in developmental pattern, reproduction, or physiology, because they co-opt entire functional networks of other genes. These evolving regulatory genes usually encode transcription factors or signaling proteins, and their evolution appears often to involve gene duplication and diversification [1].

Nearly half of all annotated transcription factors in the human genome belong to the C2H2 zinc finger (ZF) superfamily [2]–[4]. Most human ZF proteins have an architecture consisting of an N-terminal domain that interacts with other proteins and a C-terminal region that consists of C2H2 (Krüppel-type) zinc finger domains that bind DNA. About 40% of human ZF superfamily members have an N-terminal KRAB domain (Krüppel-Associated Box), which can confer transcriptional repression by recruiting KAP-1, which in turn recruits histone deacetylase and histone methyltransferase machinery to effect chromatin modification and gene silencing [5]–[17]. The KRAB domain may also be important in epigenetic gene silencing [18],[19]. Many ZF proteins contain multiple tandem C2H2 zinc finger motifs; we will refer to these as poly-ZF proteins to distinguish them from proteins with only a few zinc fingers, often not found in tandem. Almost all KRAB-ZF proteins fall into the poly-ZF category, in addition to many proteins with several tandem C2H2 repeats but no KRAB domain. The number of zinc finger repeats in poly-ZF proteins varies in humans from 4 to more than 30, with a mean of about 8.5. Most of the ZF repeats in these proteins are present in tandem and they are remarkably homogeneous in their spacing and core structure: nearly all are 21 amino acids long with the pattern C-X_2_-C-X_12_-H-X_3_-H, and they are separated from each other by a 7 amino acid linker of conserved sequence.

Despite their prevalence, the organismal functions of members of the KRAB-ZF family are remarkably obscure. For example, only three KRAB-ZF genes have been associated with human disease. Specifically, mutations in any of three closely-related KRAB-ZF genes on Xp11 may cause nonsyndromic X-linked mental retardation (see ZNF41/OMIM314995/MRX89, ZNF81/OMIM30498/MRX45, ZNF674/OMIM300573/MRX92) [20]–[22]. In some proteins, other N-terminal domains appear instead of or alongside the KRAB domain; SCAN [23] and BTB/POZ [24],[25] domains are most common (reviewed in [26]).

These properties are unusual from both functional and evolutionary perspectives. Tandem zinc fingers can bind tandem sets of 3 nucleotides, with most of the sequence-specificity for the binding of each DNA triplet contributed by the corresponding protein finger [27]. Consequently, the average human poly-ZF protein has the potential to bind sequences up to 25 to 26 nt in length (8.5 ZF repeats), far longer than typical transcription factors. The longest human poly-ZF proteins could specifically bind at sites of 80 to 90 nt in length. Since a 16 nt sequence appears on average once in a random 3 Gb genome, even accounting for substantial degeneracy in binding these very long potential recognition sites are surprising. One possibility, consistent with previous evidence, is that proteins containing large numbers of tandem C2H2 zinc fingers do not use all fingers to bind one specific target but rather bind several different targets using different subsets of their zinc fingers (reviewed in [28]).

Despite the large number and diversity of poly-ZF proteins encoded in the human genome, a large poly-ZF gene family seems to be a recent invention. The ancestral size of the poly-ZF gene family is small, and the addition of the KRAB domain as a transcriptional repressor first arose in the tetrapod vertebrates [5],[29]. Additionally, the KRAB-ZF proteins have been recognized as important subjects of lineage-specific expansion in vertebrates [30]–[32]. Rapid expansion of this gene family has occurred on the primate lineage, and a substantial proportion of human poly-ZF genes have no mouse ortholog. It has been speculated [33] that poly-ZF proteins could be important high-level regulators of transcription in primates, and could be the source of some of the major transcriptional differences between humans and other apes and within human populations. Here, we analyze the evolutionary origins of the mammalian poly-ZF family by systematically identifying poly-ZF genes in a set of diverse eukaryotes and we investigate the role of positive Darwinian selection in the expansion and functional diversification of the family in mammals.

## Results

### Sequence Collection and Characterization of Poly-ZF Proteins

Potential members of the poly-ZF family from human, mouse and 19 other species were defined and collected as described in Methods. There is no single good criterion for assigning a protein as a member of the poly-ZF gene family, so we ascertained a set that is probably larger than the true poly-ZF gene family but should contain all true poly-ZF proteins.

**Figure 1:**
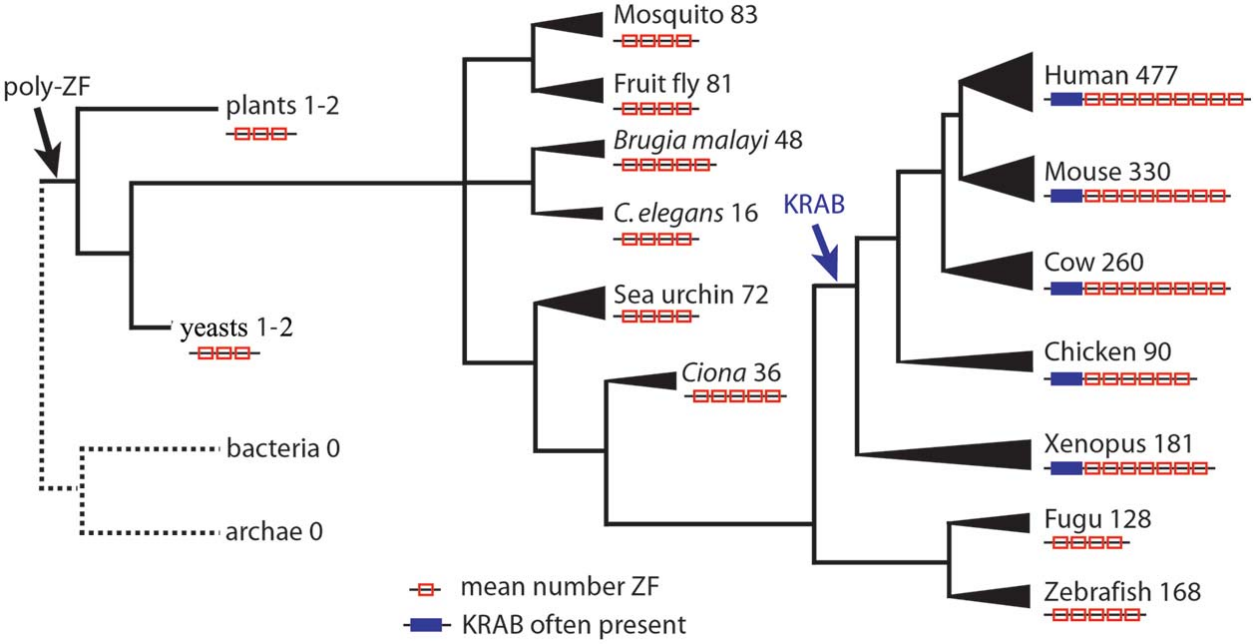
The Poly-ZF Gene Family Across Species. Graphical summary of the poly-ZF gene family in several species and groups, arranged roughly in order of divergence from human. Groups analyzed were as follows: bacteria and archaea (entire RefSeq27 protein set for each), plants (*O. sativa*, *A. thaliana*), yeasts (*S. cerevisiae*, *S. bayanus*, *S. castellii*, *S. kluyveri*, *S. mikatae*), Mosquito (*A. gambiae*), Fruit Fly (*D. melanogaster*), *Brugia malayi*, *C. elegans*, Sea Urchin (*S. purpuratus*), Sea Squirt (*Ciona intestinalis*), Fugu (*T. rubripes*), Zebrafish (*D. rerio*), Xenopus (*X. tropicalis*), Chicken (*G. gallus*), Cow (*B. taurus*), Mouse (*M. musculus*), Human (*H. sapiens*). The number next to the name of each species or group indicates the number of proteins per species with at least three tandem ZF repeats. The width of the branch leading to each group is proportional to the number of proteins to give a graphical indication of relative gene family size. The cartoon under each name indicates the mean number of tandem ZF repeats in proteins that contain at least three tandem ZF repeats. A blue box is added to the N-terminal end of each cartoon if that group contains any KRAB-ZF proteins.

The zinc finger repeats in most human and mouse poly-ZF proteins are located on a single large exon; N-terminal domains are usually present on small exons, often separated from the zinc finger exon by large introns [33],[34]. Because of this structure and the appearance of several distinct N-terminal domains in poly-ZF proteins, inclusion of any particular N-terminal domain in gene predictions may not be a reliable indicator of gene relatedness. Family membership was instead defined by the presence of multiple tandem zinc finger domains (see Methods). Assuming similar annotation qualities among genomes, the human genome contains a substantially larger number of genes than mouse, and both the human and mouse genomes contain more poly-ZF proteins than any of the other species analyzed (Table 1). The ancestral size of the family in chordates is small (Figure 1), so it is likely that the large number of human genes results from expansion of the family along the primate lineage. Judging from protein relatedness among paralogs, gene duplications contributing to the expansion have occurred irregularly over time, with no obvious bursts of duplication. Among human and mouse poly-ZF proteins, a wide range in the number of zinc finger repeats is observed; the mean number of zinc finger repeats in human proteins is 8.5, but some proteins contain 30 or more zinc finger repeats. Mouse poly-ZF proteins contain an average of 7.5 zinc finger repeats each; several mouse proteins contain more than 20 zinc finger repeats.

**Table 1:** Poly-ZF Family and Domain Summary.

Species	Poly-ZF	BTB	KRAB	SCAN
**Human**	712	50	304	53
**Mouse**	583	44	219	38
**Cow**	482	41	106	28
Dog*	329	41	61	17
**Chicken**	224	26	33	0
**Xenopus**	347	30	21	0
Zebrafish*	405	46	0	0
**Takifugu**	364	41	0	0
**Drosophila**	251	11	0	0
**Anopheles**	263	9	0	0
**Ciona**	103	7	0	0
**C. elegans**	108	1	0	0

For each species analyzed, this table shows the number of poly-ZF proteins identified in that species (see Methods for criteria). In addition, the number of BTB, KRAB, and SCAN domains detected in poly-ZF proteins is shown, based on searches of the PFAM database. * tblastn searches of the genome suggest that the number of poly-ZF genes is greatly underestimated in these two cases.

The PFAM database was used to detect the presence of additional N-terminal domains in poly-ZF proteins from many species. Table 1 summarizes both the number of poly-ZF matches in each of 12 species and the counts of common N-terminal domains. Our criteria for inclusion were liberal in order to ensure a high sensitivity in detecting poly-ZF genes, so these counts are likely overestimates of the number of intact functional members of the poly-ZF family. In addition, the quality of gene prediction and annotation in many species makes an accurate estimate of the true number of poly-ZF proteins difficult. Gene counts range from just over 100 in *Ciona intestinalis* to 712 in human, with most animal species having several hundred genes, and mammals tending to have more genes than non-mammals. The basic domain structure of poly-ZF genes appears to have arisen near the root of eukaryotes (Figure 1, Figure S1). This result is consistent with previous work indicating significant expansion of the KRAB-ZF protein family in tetrapods [31]. Blastp alignment scores were used to estimate the proportion of poly-ZF genes with a closely related paralog in each gene family. Since paralogs begin with identical or nearly identical sequences and diverge over time, we infer that the presence of many genes with closely related paralogs implies substantial recent gene duplication. Evidence of recent duplication by this method correlates well with our estimated gene counts: species estimated to have more poly-ZF genes also have stronger evidence for recent duplications, with humans having the most recent duplication activity (Figure S2). As previously reported, the BTB domain is found associated with poly-ZF genes in all animal species considered, whereas the KRAB domain is restricted to the tetrapod vertebrates, and the SCAN domain is restricted to mammals [5],[23],[26],[29]. Our method assigned poly-ZF family membership to 53 SCAN-ZF proteins, which is in good agreement with a previous estimate of 58 SCAN-ZF proteins in the human genome [35]. Many predicted genes in all species analyzed contained no annotated N-terminal domains. This lack of N-terminal domains could represent proteins with only tandem ZF domains, or it could be due to incomplete annotation failing to assign small distant N-terminal exons, the presence of pseudogenes with N-terminal deletions, or the presence of novel unannotated N-terminal effector domains.

Figure 1 shows a schematic of the poly-ZF gene families present in many diverse eukaryotic species, detailing for each species or group of species the number of proteins that contain at least three tandem ZF repeats of canonical structure, the mean number of ZF repeats among these proteins, and the presence or absence of KRAB domain-associated poly-ZF proteins. The figure shows the small ancestral size of the poly-ZF gene family and demonstrates the rapid expansion of both poly-ZF gene number and the number of ZF repeats per gene on the vertebrate lineage.

### Gene Expansions and Chromosomal Clustering in Human and Mouse

In order to detect probable lineage-specific expansions in the species analyzed, sets of paralogous poly-ZF genes were identified in each species. Briefly, the neighbor-joining method on a pairwise similarity measure was used to cluster all poly-ZF genes for five sets of two species each: human and mouse, cow and dog, chicken and frog, fugu and zebrafish, and mosquito and fruit fly. Using this method, multiple proteins from one species clustering together without any proteins from the sister species is taken as evidence of paralogy. All five clustering trees are characterized by species-specific clades of closely-related paralogs interspersed with many orthologous pairs of genes. Lineage-specific expansions were defined as clades including at least 5 proteins from one species that cluster together (see Methods, Figure S3). Lineage-specific expansions are common; for example, when human and mouse sequences were compared, 17 expansions specific to human and 7 expansions specific to mouse were found. The largest human lineage-specific expansion contained 33 genes, and the largest mouse lineage-specific expansion contained 22 genes. In total, 157/712 human genes (22%) and 85/583 mouse genes (15%) were contained within lineage-specific expansions by our criteria.

**Figure 2:**
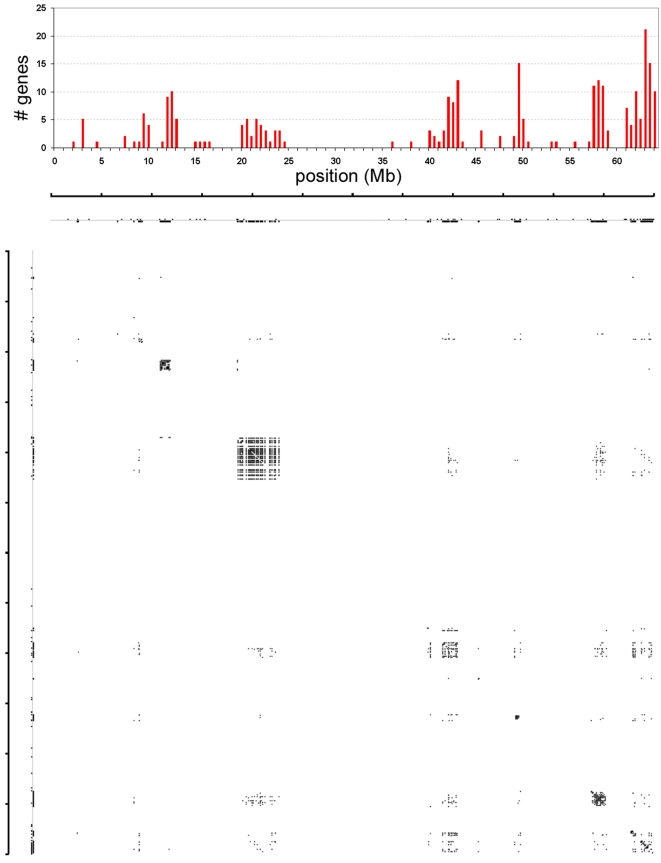
Poly-ZF Genes on Human Chromosome 19. Top panel: histogram of the number of poly-ZF genes in each 500 kb bin along chromosome 19. Lower panel: dot-plot of identity between poly-ZF proteins. Each dot represents a pair of genes with at least 59% identity. Darker spots represent pairs of genes with higher identity scores. Box-like patterns indicate high similarity between proteins encoded by genes in the same physical cluster.

Poly-ZF genes are strongly clustered in the human genome, consistent with a history of local gene duplication and divergence [32],[34]. For example, 37% of human poly-ZF genes (263 of 712) are located on chromosome 19, mostly in 6 large gene clusters, consistent with previous results indicating a high number of lineage-specific gene family expansions on human chromosome 19 [36]. The largest cluster of human poly-ZF genes on chromosome 19 spans ∼3.5 Mb and contains 72 poly-ZF genes. Figure 2 plots the locations of poly-ZF genes on human chromosome 19, grouped into 500 kb bins. The lower part of the figure shows a 2-dimensional plot of similarity among the human poly-ZF proteins with chromosome position on both axes. Dots on the axes represent the locations of poly-ZF genes. Dots inside the plot represent blastp matches between genes with at least 59% AA identity (chosen for visual clarity), with self-matches excluded and darkness proportional to sequence identity. The strong box patterns indicate local similarity: proteins from the same gene cluster tend to be more similar than those that are not located in close physical proximity. For example, almost all of the proteins encoded by genes in the large poly-ZF cluster at 20–25 Mb are very similar to all other genes in the cluster, but matches to the other poly-ZF genes are more divergent. A permutation test indicates that the correlation between genomic proximity and high sequence identity is highly significant (Figure S4). These patterns are consistent with a simple duplication process in which new genes arise largely by local duplication and gradually disperse by subsequent genome rearrangements.

### Positive Selection in Lineage-Specific Expansions

To determine what role adaptive molecular evolution has played in the expansion and diversification of poly-ZF genes, we assessed signatures of positive selection among recently duplicated genes. Maximum-likelihood analysis of codon evolution was conducted on closely-related groups of genes across the entire sets of mouse and human poly-ZF genes, and on the largest lineage-specific expansions in the cow, chicken, fugu, frog, fruit fly, mosquito, and zebrafish poly-ZF families. Lineage-specific expansions were collected as described above; expansions of 5 or more genes were chosen to produce codon diversity sufficient for powerful maximum-likelihood analysis [37],[38]. Table 2 summarizes results of d_N_/d_S_ analysis. The number of poly-ZF sequences and p-value for positive selection are shown for the lineage-specific expansion from each species with the best evidence for positive selection. Complete results of codeml analysis on all species can be found in Dataset S1. To test the robustness of these results, analysis was repeated using the more conservative codeml M1a versus M2a likelihood ratio test, and results were essentially the same (data not shown). Misalignment of sequences and recombination among sequences can be problematic when using these methods. Misalignment is unlikely to be a significant problem, since the zinc finger domains within each poly-ZF protein have substantial amino acid sequence diversity, with patterns similar to those observed for zinc finger repeats as a whole (Figure S5). Visual inspection of alignments also supported their validity. To investigate the possibility of recombination events, the program GENECONV was used to detect gene conversions among paralogous genes. A few genes in many of the sequence sets showed evidence of short regions of gene conversion. Datasets were reanalyzed with all genes showing evidence of a gene conversion removed, and similar results indicating extensive positive selection were obtained. One exception was the two sets of genes from chicken, where most genes showed evidence of gene conversion events; the evidence for positive selection in these cases should thus be interpreted with caution.

**Table 2:** Evidence for Positive Selection.

Species	# genes	p-value
*A. gambiae*	22	N/S
*B. taurus*	9	1.29E-49
*D. rerio*	33	9.63E-10
*D. melanogaster*	25	N/S
*G. gallus*	14	4.32E-8
*H. sapiens*	33	3.35E-82
*M. musculus*	22	7.47E-24
*T. rubripes*	27	3.58E-15
*X. tropicalis*	11	2.06E-22

Next to each species is shown the number of genes analyzed, along with the p-value for the detection of positive selection using the M7 vs. M8 LRT implemented in codeml. Each analyzed set of genes is from a single lineage-specific poly-ZF expansion from that species (see Methods). Only the expansion with the best p-value from each species is listed here; significant p-values indicate adaptive evolution (d_N_/d_S_>1).

Many of the groups of genes included in human and mouse lineage-specific expansions showed strong evidence of positive selection (p<0.05), with 71% of all expansion proteins falling into groups in this category. Those human and mouse expansions that did not show evidence of adaptive evolution were among the smallest; lack of evidence for positive selection in these groups could be the result of the lack of power incurred when the likelihood ratio test is used on small sequence sets [37],[38]. Signals of positive selection could also be obscured by long periods of purifying selection following gene duplication and diversification. Table S1 gives a summary of the tests for positive selection in each of the 24 mouse and human gene expansions analyzed. Some lineage-specific expansions from cow, frog, fugu and zebrafish all showed strong evidence for positive selection. In contrast, poly-ZF expansions from *D. melanogaster* and *A. gambiae* showed no evidence for positive selection. Many of the largest gene expansions from each species gave huge differences in maximum-likelihood between the neutral and positive selection models (Table 2), indicating strong positive selection and statistically robust results.

### Selection Sites and Zinc Finger Structure

Deducing the specific selective pressures that have acted on poly-ZF gene family members requires identification of the position and function of the amino acids under positive selection. X-ray crystal structures are known for several C2H2 zinc finger proteins bound to DNA, including five distinct zinc fingers with the residue spacing and finger linker sequence that characterize the KRAB-ZF family [39],[40]. The main nucleotide specific contacts in these crystal structures are made by amino acids at positions −1, 3, and 6 of the zinc finger α-helix, with positions 1 and 2 contributing contacts in some zinc fingers [27],[41]. It should be noted that in conventional nomenclature there is no position 0, so positions 1 and −1 are adjacent. To test whether positive selection in the poly-ZF family is likely to affect DNA binding specificity, the Bayes Empirical Bayes method implemented in codeml was used to determine specific sites subject to probable positive selection among each set of genes that showed significant evidence for positive selection [42],[43]. Patterns of these sites were striking and remarkably similar across all groups: sites of positive selection were almost entirely restricted to the zinc finger domains and were heavily concentrated in specific residues within the fingers. The specific residues with the strongest and most consistent evidence of positive selection were the three amino acids known to form the main nucleotide specific contacts in characterized DNA-binding zinc fingers [27]. Sites of positive selection were usually distributed across many or all of the zinc fingers with no obvious pattern other than concentration in the major nucleotide specificity residues.

**Figure 3:**
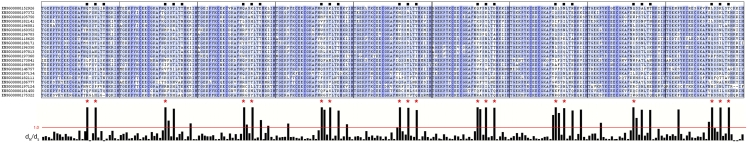
Example Poly-ZF Alignment. A protein multiple alignment of the largest human poly-ZF gene expansion, with some sequences removed for clarity (see Methods). Columns with any gaps have been removed. The 9 zinc fingers included in the alignment are outlined in black boxes, and black squares mark the three major nucleotide specificity residues (positions −1, 3 and 6) in each zinc finger. The darkness of the blue background is proportional to amino acid conservation. Below is plotted the posterior mean d_N_/d_S_ value assigned by Bayes-Empirical-Bayes analysis at each position. The red line represents d_N_/d_S_ = 1 (no selective pressure), and red stars mark residues at which the hypothesis d_N_/d_S_>1 reaches statistical significance (P> = 0.95).

**Figure 4:**
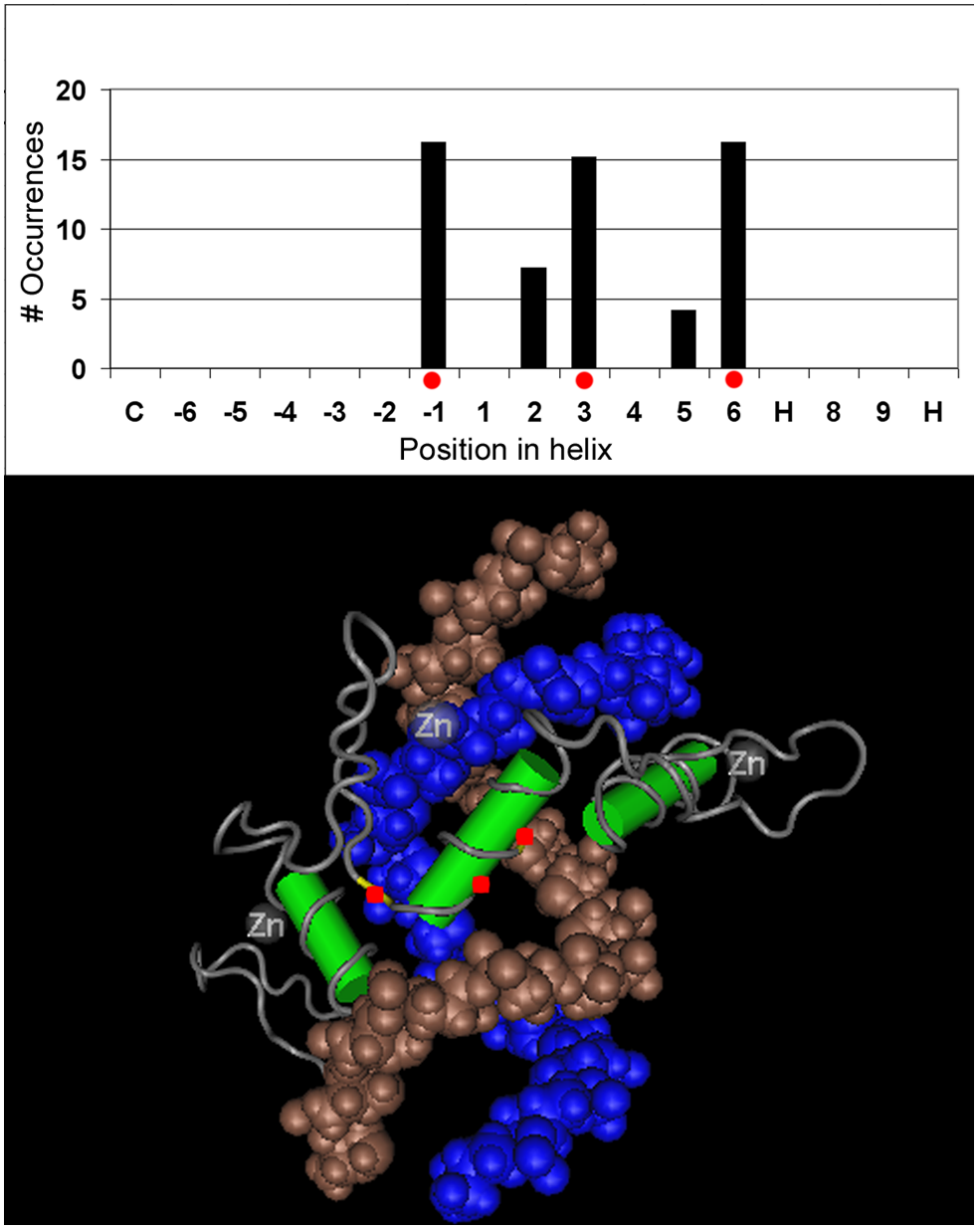
Selection Sites and ZF Structure. The top panel shows the number of times each position in the ZF α-helix was found to have significant evidence for positive selection by Bayes-Empirical-Bayes analysis. Data were gathered from the largest poly-ZF expansion from each species analyzed (see Methods). The lower panel shows the crystal structure of the first three zinc fingers of *Xenopus laevis* TFIIIA bound to DNA [61]. Structure is visualized using Cn3D software (http://www.ncbi.nlm.nih.gov/Structure/CN3D/cn3d.shtml). In both parts of the figure, the primary nucleotide-contact residues (−1, 3 and 6 in the helix) are marked by a red dot. On the bottom panel, positions −1, 3 and 6 are ordered from left to right.

As an example, Figure 3 shows a segment of multiple alignment from the largest human poly-ZF expansion. Nine zinc fingers are aligned; each aligned finger includes at least one site of positive selection, and all 19 sites identified as being under positive selection are at the major nucleotide specificity positions −1, 3 or 6. In addition, most of the other major nucleotide specificity positions have a best d_N_/d_S_ estimate over 1.0, though they did not reach statistical significance. Figure 4 summarizes the frequency of positive selection at each site in the zinc finger DNA-binding α-helix among all the largest poly-ZF expansions from each species analyzed, along with an X-ray crystal structure of a similar zinc finger. The crystal structure illustrates the position of the three major nucleotide specificity residues with respect to the bound DNA molecule. Positively selected sites are concentrated in the major nucleotide specificity residues, with a lower frequency of sites at adjacent residues (positions 2 and 5). This pattern suggests that adaptive evolution in poly-ZF gene expansions has acted mostly to change DNA binding specificity. This conclusion is consistent with previous results indicating elevated dN/dS in recent human paralogs and a bias toward mutations at positions −1, 3 and 6 suggestive of changes in DNA binding specificity, although previous work involved only subsets of human poly-ZF genes [44],[45].

**Figure 5:**
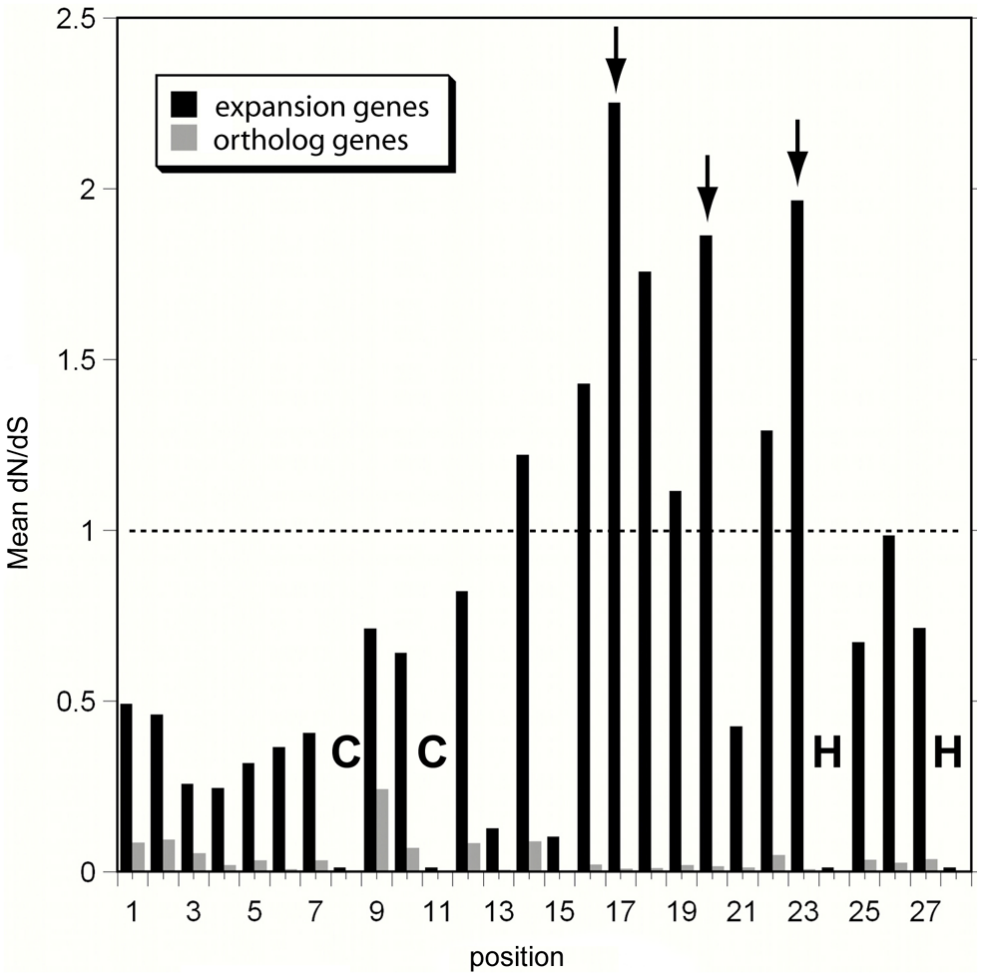
d_N_/d_S_, Orthologs vs. Expansions. Plot of the value of d_N_/d_S_, averaged over expansion or orthologous fingers. Values for expansion genes are derived from 131 genes from all human and mouse poly-ZF expansions. These genes comprise 25 alignment groups representing 127 gap-free C2H2 zinc fingers. Ortholog values are calculated over 411 genes from 137 sets of mouse, human and cow orthologs, representing 1149 gap-free C2H2 zinc fingers. Each zinc finger site in each alignment was assigned its peak d_N_/d_S_ value from the 11-class BEB output, and the average of this value over all alignments is plotted. Arrows indicate major nucleotide specificity residues. The systematically higher values among expansion fingers result from a shift in assigned codeml dN/dS classes due to the positively selected sites; they do not indicate relaxed negative selection.

As summarized in Figure 5, the pattern of conservation of zinc finger residues is dramatically different among orthologous poly-ZF genes. In contrast to patterns among recently duplicated genes, among 1∶1∶1 cow-human-mouse ortholog trios all residues in the DNA binding α-helix are highly conserved. This result indicates that evolutionarily stable orthologs in the poly-ZF gene family are under strong purifying selection to retain their respective DNA binding specificities, underscoring the peculiar properties of recently duplicated poly-ZF genes.

### Evolution by Zinc Finger Gain and Loss

In addition to point mutations, which supply the raw material for the adaptive evolution investigated by d_N_/d_S_ methods, the gain and loss of entire zinc finger domains, presumably mediated by recombination, could enable rapid functional divergence among recently duplicated paralogs. Especially in cases in which tandem zinc fingers bind a single DNA target site, a single insertion or deletion of a zinc finger domain could drastically alter the binding target of a poly-ZF protein.

To investigate the effect that zinc finger gain and loss could have on the functional divergence of poly-ZF paralogs, the number of intact canonical zinc finger domains was measured for the poly-ZF proteins from each mouse and human lineage-specific expansion identified above and in 137 trios of orthologous mouse-cow-human poly-ZF proteins. Genes for which there was significant evidence of gene conversion were excluded. Of the 137 ortholog trios, the genes from all three species had the same number of zinc finger domains in 111 cases. Among the 24 lineage-specific expansions from human and mouse that were examined only one group of two paralogs had the same number of zinc fingers. The median standard deviation in zinc finger number was 1.94 for sets of paralogous proteins (see Dataset S1). Changes in zinc finger count can be caused by the deletion or duplication of whole zinc fingers or by mutational inactivation, particularly small indels and mutations of the C and H resides that coordinate zinc binding. Inspection of specific paralog alignments indicated that deletion or duplication of whole zinc finger domains was the most common source of the observed differences. The higher frequency of change in zinc finger number among paralogs is particularly striking since the paralog sets have undergone duplication and divergence since the separation between Glires (which includes rodents) and Primates and are therefore more recent than the orthologous genes. These results suggest that the duplication, deletion and inactivation of zinc finger domains has been an integral part of the functional diversification of mammalian poly-ZF proteins.

### Zinc Finger Number and Diversity Counts

**Figure 6:**
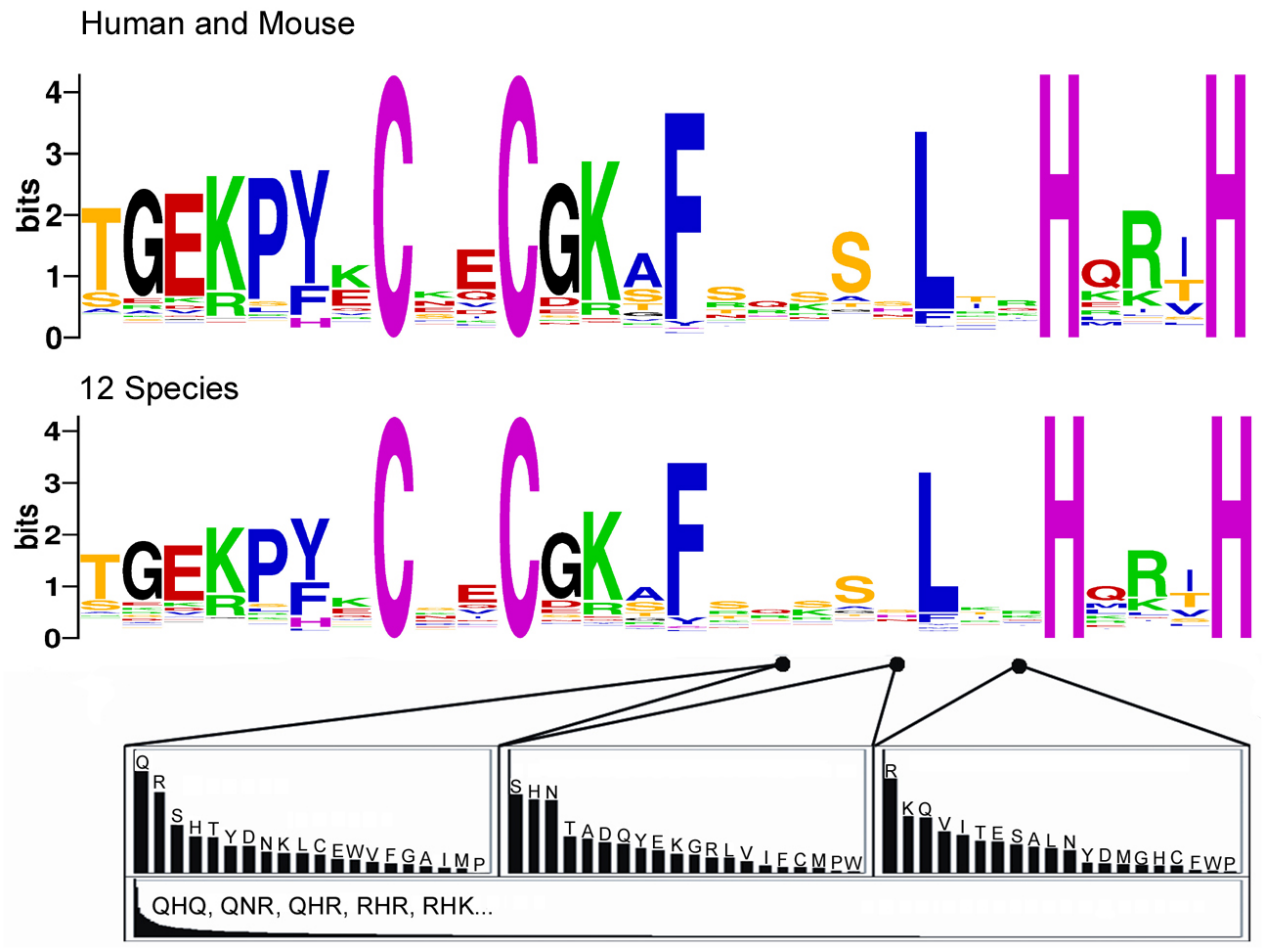
Zinc Finger Diversity. Above: a logo representation of amino acid diversity among 9,737 zinc fingers collected from human and mouse poly-ZF proteins. Below: a logo constructed from 23,797 zinc fingers collected from the poly-ZF proteins of all major species analyzed. A high bit score on the logo plot reflects invariant residues (C, C, H and H are ascertained to be invariant and so provide a scale for reference). The logo plots are nearly identical, representing the deep conservation of the C2H2 zinc finger motif. Below the logo plots are three charts detailing the amino acid diversity at each of the three major nucleotide specificity residues in the zinc finger. Columns are the frequencies of each amino acid, sorted from highest to lowest at each position. At the bottom is a chart of the frequencies of each triplet of amino acids obtained by combining the residues at −1, 3, and 6, sorted from highest to lowest. The five most frequent triplets are listed in order. See supplemental materials for a table of the amino acid counts and frequencies at each position and for all triplets.

Another view of sequence diversity in zinc fingers from poly-ZF genes was obtained by simply collecting all of the canonically spaced zinc fingers (C-X_2_-C-X_12_-H-X_3_-H) from all poly-ZF genes in the 12 main species analyzed (see Methods). Figure 6 shows a logo representation of sequence diversity among these fingers. Underneath the logo plot are schematics detailing the diversity of amino acids at the three major nucleotide specificity residues, and for the three-amino-acid combinations of these residues. As expected, several residues in the zinc finger sequence are well conserved, including residues involved in zinc coordination and in non-specific DNA phosphate contacts, and the 7-amino acid linker sequence that separates tandem zinc fingers. In contrast, the three major nucleotide specificity residues stand out as having high amino acid diversity. Among the total collection of 23,797 C2H2 zinc fingers, 3,268 of the 8,000 possible amino acid triplets are represented at least once, but the top 10% of the observed amino acid triplets account for 50% of the observed zinc fingers. Despite the remarkable amino acid diversity observed at the major nucleotide specificity residues, the amino acid frequencies at each of the three positions are distinct from each other and distinct from the universal protein average (Table S2). This result indicates that each position is subject to specific selective constraints, presumably related to compatibility with making productive nucleotide contacts and a stable folded structure.

## Discussion

### Evolution of the Poly-ZF Family

Genes containing several tandem C2H2 zinc fingers with the spacing characteristic of the poly-ZF family appear to have arisen first near the root of the eukaryotic lineage. No such genes were found in the entire bacterial and archaeal protein set, but examples were found in several different plant and fungal genomes. In plants and fungi the number of poly-ZF genes and the number of zinc fingers within each gene are very small. We presume that a small number of poly-ZF genes with few zinc finger domains represents the ancestral state of the poly-ZF gene family. The presence of large families of genes with many tandem fingers is restricted to metazoans, suggesting that the family began to expand in both gene number and number of fingers around that time. Most poly-ZF genes have one large tandem ZF array – multiple smaller ZF arrays are rarely seen (Figure S6), suggesting that this unique domain architecture evolved early in the expansion of the gene family. Addition of the KRAB and SCAN domains was roughly concurrent with an even more dramatic poly-ZF gene family expansion in the tetrapods, and is most extreme in the mammalian lineages [5], [30]–[32]. Nevertheless, lineage-specific expansion in the poly-ZF family is limited neither to mammals nor to proteins with KRAB domains: for example the fugu and zebrafish poly-ZF genes do not contain any KRAB domains, but both show evidence for lineage-specific expansions.

Many mouse and human poly-ZF genes are located in gene clusters, and genes that share physical proximity tend to be more closely related in sequence. There are also no obvious peaks in the degree of divergence of existing duplicate genes. These observations suggest that the primary mechanism underlying gene family expansion has been sporadic local duplication events affecting one or a few genes, followed by slower genomic rearrangements that break up gene clusters over time. Some of the discrepancy in gene number among species could be due to gene loss in particular lineages, but the overall trend has probably been one of gene family expansion. The presence of gene family expansions in divergent lineages suggests that the poly-ZF gene family provides a framework for rapid transcriptional evolution that has been utilized repeatedly.

### Biochemical Function

Members of the poly-ZF protein family in humans and other mammals are thought to bind DNA via their zinc fingers and to drive local chromatin toward the closed state via their KRAB, SCAN, BTB, or SET domains [14]–[19], [23]–[26]. An unsolved problem is why most poly-ZF proteins have so many zinc fingers and why the evolution of the family in many organisms shows a trend toward an increasing number of ZF repeats. This trend implies a selective force acting to expand the number of tandem ZF domains, but the nature of this selective force remains unclear. The expansion could in part be due to a need to retain unique binding sites in growing genomes, but this seems unlikely to explain the whole effect: many mammalian poly-ZF proteins have far more ZF repeats than should be necessary to bind to a single unique sequence in their respective genomes. Another possibility is that many poly-ZF proteins contain more than one DNA binding domain, i.e. long tandem ZF arrays may be functionally broken into several regions of fewer ZF repeats, each of which binds its own specific site in the genome. Under this model, the poly-ZF gene family has evolved to target a very large number of DNA binding sites by expanding both the number of poly-ZF genes and the number of binding domains within each gene. Each of these binding domains could independently initiate a standard program of transcriptional repression by driving local chromatin toward a closed state. Regardless of the ultimate cause, the pattern of expanding tandem ZF arrays agrees with our positive selection results in suggesting that much of the selective pressure acting in the family is related to their DNA binding activity. Our results indicating that the expansion and contraction of zinc finger domain arrays is common among paralogous genes suggests that these changes contribute to DNA-binding plasticity. Gain and loss of whole zinc finger domains by recombination-based mechanisms would probably result in a fully functional protein with substantially altered DNA-binding specificities, and could allow for very rapid divergence in the binding properties of new paralogs.

### Organismal Function

The number of poly-ZF genes in the human genome and the recent expansion and adaptive evolution in parts of the family indicate that some of these genes have played some important role in the evolution of the lineage leading to humans. Despite this, there is remarkably little information about the ultimate *in vivo* function of poly-ZF genes, and what evidence exists does not point in any clear direction. Three closely-related human genes are implicated in non-specific X-linked mental retardation (see ZNF41/OMIM314995/MRX89, ZNF81/OMIM30498/MRX45, ZNF674/OMIM300573/MRX92) [20]–[22], although the evidence in these cases is not entirely conclusive. Another well-studied poly-ZF protein is CTCF, which is known to bind several different targets in the genome and to function as a transcriptional insulator by inducing chromatin remodeling [46]–[48]. ZFP206, a human SCAN-ZF gene located in a gene cluster on Chr. 16, has been implicated in the maintenance of embyronic stem cell pluripotency [49]. Rsl, a mouse KRAB-ZF protein, is known to influence sexually dimorphic gene expression in the liver [50]. It has been suggested that OTK18, a human poly-ZF protein on Chr. 19, is important in transcriptional repression of the HIV long terminal repeat promoter activity [51]. Recently the *chato* mutation, in a mouse KRAB-ZF gene, has been found to cause defects in convergent extension during development [52].

Though it seems likely that most or all of the poly-ZF genes act as transcriptional regulators, the dichotomy between orthologous poly-ZF genes and expanded gene clusters, in addition to the wide range of functions attributed to previously studied poly-ZF proteins, suggests the possibility of more than one type of organismal function. One or more functions, associated with highly conserved orthologous genes, must be relatively stable in mammals. For example, these may be regulators of development that specifically target suites of genes in a coordinated fashion as development unfolds. Another function (or set of functions), associated with the genes undergoing duplication and diversification, is likely to be subject to evolutionary pressure to expand gene and finger number and to change DNA binding specificity.

We hypothesize that the modular structure of poly-ZF genes forms an ideal substrate for rapid evolution of transcriptional regulation. With the possibility of multiple sequence-binding domains made up of groups of zinc fingers coupled to a transcriptional repression domain that operates independently of sequence target, only a handful of point mutations or small rearrangements would be necessary to effect a significant change in the transcriptional state of multiple target genes. This flexibility may make the unstable subset of the poly-ZF gene family well suited to rapid adaptive evolution of transcriptional patterning. One possibility is that they function to repress transcription of viral or transposon genes and that their evolution is driven by an arms race with their viral or transposon targets. Alternatively, they may contribute to rapid morphological and behavioral evolution by modulating transcription of developmental genes. Once target DNA binding sites are known for many poly-ZF genes, tests of co-evolution between poly-ZF genes and their targets could help shed light on the evolutionary forces underlying the rapid expansion and diversification of zinc finger genes. In addition, population genetic studies could help distinguish between positive selection and selection to maintain diversity as mechanisms influencing the evolution of this gene family. Elucidation of the organismal function of recently expanded human poly-ZF genes will be an important step toward a further understanding of recent primate evolution and could be important in understanding modern human disease and genetic variation.

## Methods

### Sequence Collection

All protein sequences predicted from NCBI human build 36 were collected from ENSEMBL BioMart. An rps-blast profile was constructed by using an artificial query of six consensus C2H2 zinc fingers to initiate a psi-blast search of all human and mouse proteins. This resulting profile was used as an rps-blast target with the entire human protein prediction set as query. All queries with an E-value below 1e-4 were kept as candidate poly-ZF family members. This list was concatenated with the list of human proteins containing at least two tandem C2H2 zinc fingers with canonical spacing, which was determined as a match to the regular expression:
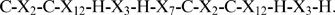



The longest splice form of each protein from the concatenated list was collected, and this was taken to be the list of human poly-ZF proteins. Recent work has produced a more carefully annotated list of some classes of human poly-ZF genes [33], but we did not use this list so as to permit valid comparison to prediction sets from other organisms.

The same approach was taken to collect poly-ZF proteins from predicted proteins sets for the following reasonably well-annotated genomes available at the time: *Anopheles gambiae* AgamP3, *Bos taurus* BTau_3.1, *Caenorhabditis elegans* WS170, *Canis familiaris* BROADD2, *Ciona intestinalis* JGI2, *Danio rerio* ZFISH6, *Drosophila melanogaster* BDGP4.3, *Gallus gallus* WASHUC2, *Mus musculus* NCBIM36, *Takifugu rubripes* FUGU4, and *Xenopus tropicalis* JGI4.1. The poly-ZF gene complements of these 12 species were used for most analysis. In addition, the poly-ZF gene complements of *Saccharomyces cerevisiae*, *Saccharomyces bayanus*, *Saccharomyces castellii*, *Saccharomyces kluyveri*, *Saccharomyces mikatae*, *Arabidopsis thaliana* TAIR7, *Oryza sativa* OS6, *Brugia malayi* BMA1 and *Strongylocentrotus purpuratus* SPUR2.1 were collected in the same way for Figure 1 but were not analyzed further. When available, the protein prediction sets were downloaded from ENSEMBL. Others were extracted from NCBI RefSeq (http://www.ncbi.nlm.nih.gov/RefSeq/) release 25 [53]. The 21 species analyzed were chosen to represent a large number of diverse higher eukaryotic lineages.

HMMer software (http://hmmer.janelia.org/) [54] was used to identify and count domains present in the poly-ZF family proteins of each of the 12 main species analyzed. All gathered candidate poly-ZF protein sequences were searched against the Pfam_ls database using HMMer version 2.3.2, and each profile hit with an E-value less than or equal to 1.0 was kept. This cutoff may result in occasional false positives but has the advantage that it sensitively detects divergent zinc fingers and N-terminal domains. The number of zinc fingers was measured as the number of hits to the PFAM (http://pfam.sanger.ac.uk/) [55] C2H2-ZF profile (PF00096) in each sequence. In addition, the PFAM search was used to determine the number of KRAB (PF01352), SCAN (PF02023) and BTB (PF00651) domains present in the poly-ZF family of each species. Many other domains were detected by PFAM, but these profiles had only one or two hits even in the largest poly-ZF families and so were subsequently ignored.

### BLAST Ratio

In order to assess the prevalence of recent duplication events in the poly-ZF gene families of the various species analyzed, we calculated a “BLAST ratio” for each poly-ZF protein in each of the four species *C. elegans*, *C. intestinalis*, *X. tropicalis*, and *H. sapiens*. For each species, an all-by-all Blastp search was performed among the poly-ZF proteins of that species. The two best Blastp hits were recorded for each query protein to compute the ratio of the score of the second-best hit to the score of the best hit (the nearest paralog match and the identity match). A high BLAST ratio indicates a protein which has a close paralog in the same species. A species with consistently high BLAST ratios indicates that the poly-ZF genes tend to have close paralogs, suggesting substantial recent duplication. Due to differences in rates of evolution across species, this should not be taken as a quantitative measure, but rather as a qualitative test of the frequency of gene duplications.

### Lineage-Specific Expansions

To detect lineage-specific expansions in human and mouse poly-ZF families, all-by-all pairwise alignment scores were computed on the concatenated set of human and mouse poly-ZF proteins using ClustalW (version 1.83). These pairwise scores were used to create a neighbor-joining tree, and this tree was used to select clades of human or mouse proteins for d_N_/d_S_ analysis. The NJ tree was manually inspected, and each clade that consisted of at least 5 sequences from either human or mouse coupled with at most one orthologous sequence from the other species was chosen for further analysis. Each of these clades was taken to represent a lineage-specific expansion of several paralogs, and the set of paralogous sequences was collected. In total, 17 clades of human sequences and 10 clades of mouse sequences were selected for further analysis. Although each clade was allowed one ortholog for selection purposes, the ortholog was not included in d_N_/d_S_ analysis. The sequence set of each lineage-specific expansion was analyzed for positive selection separately. Three mouse clades were removed because their ungapped multiple alignments covered one zinc finger repeat or less, leaving 7 mouse clades for analysis. This process was repeated to generate lists of lineage-specific expansions for the following pairs of species: *Anopheles gambiae & Drosophila melanogaster*; *Danio rerio & Takifugu rubripes*; *Gallus gallus & Xenopus tropicalis*; *Bos taurus & Canis familiaris*. No lineage-specific expansions in *C. familiaris* relative to *B. taurus* were found using this method, probably due to an incomplete *C. familiaris* prediction set for poly-ZF genes (data not shown). All lineage-specific expansions from each species were collected and tested for positive selection. In some cases, a particularly large species-specific clade was broken into several smaller sets for analysis due to the computational time and optimal tree length required by codeml.

### Chromosomal Clustering

Biomart (www.biomart.org) was used to identify the genomic locations of all human poly-ZF genes. Since human Chr. 19 contains the most poly-ZF genes, these genes were chosen for analysis. The genes were placed along the length of human Chr. 19 in 500 kb bins, with location determined by start position of the longest splice form. For dotplot analysis, GenoPix 2D [56] software was used. Briefly, an all-by-all BLAST comparison was performed among the poly-ZF genes on human Chr. 19, and matches with >59% identity were plotted, with darker dots corresponding to alignments of higher identity. The percent identity cutoff was arbitrary and chosen to present the clearest visual output.

To assess the significance of the result that protein similarity is higher within than between poly-ZF gene clusters on human Chr. 19, a random permutation test was performed. Pearson's r was calculated between the genomic distance and % amino acid identity of all pairwise combinations of poly-ZF genes to determine the real position-identity correlation. To test significance, 25,000 trials were carried out in which the % amino acid identity for each pair of poly-ZF proteins was kept intact but the locations of the 263 poly-ZF genes on chromosome 19 were randomized without replacement.

### Positive Selection

Positive selection among selected sequences was measured using the codeml program from the PAML software package (version 3.14) [42],[43]. The Bayes Empirical Bayes method implemented in codeml has been shown to work well on real datasets, and has both good power to detect amino acid sites under positive selection and a very low false positive rate on simulated datasets [43]. Briefly, for each set of sequences to be analyzed, a multiple protein alignment and sequence tree were created using ClustalW (www.ebi.ac.uk/clustalw/) [57] and Bonsai (http://depts.washington.edu/jtlab/software/softwareIndex.html) software. Each protein sequence multiple alignment was used as a guide to produce the corresponding codon multiple alignment. The codon multiple alignment and tree for each set were used as inputs to codeml. Codeml runs were performed with model 7 (which models site omega classes as a beta distribution with 0≤ω≤1) and model 8 (model 7 plus an additional site class at omega ≥1). Significance was assessed by comparing twice the difference in log-likelihood between models 7 and 8 to a χ^2^ distribution with 2 degrees of freedom. Each model 8 run was performed three times, with starting ω = 0.3, 1.0, and 3.4 to avoid problems resulting from local optima. The Bayes Empirical Bayes method implemented in codeml was used to determine specific sites of probable positive selection, using the M7-M8 comparison. The threshold used for significance in this case was p(ω>1)≥0.95. Models M1a and M2a were run on the same datasets as M7 and M8, and the same test of significance (2* ΔML compared to χ^2^ with 2 degrees of freedom) was used. The program GENECONV was used to detect gene conversions [58]. GENECONV was run with default parameters and all sequences involved in a gene conversion with simulation-based P-value<0.01 were removed prior to rerunning codeml using models M7 and M8.

### Intrafinger Alignments

In order to explore the possibility of concerted evolution within genes, multiple alignments of the zinc finger repeats from each human poly-ZF protein with exactly 18 zinc fingers were created. A protein multiple alignment was made using all the zinc fingers from a specific gene, including the 7 amino acid linker sequence. The amino acid logo was created from a collection of 4,113 zinc finger repeats from human poly-ZF proteins using WebLogo software (http://weblogo.berkeley.edu) [59].

### Ortholog and Expansion dN/dS

137 sets of orthologs from human, mouse, and cow were compared to sets from all large human and mouse specific gene expansions. For each set, a protein alignment was used to guide a codon alignment for the corresponding coding mRNA. Individual aligned fingers were extracted and all fingers with gaps were removed from the analysis. codeml was run on each set of aligned fingers using model 8 (starting ω 1.0). For each finger position, the values of the maximum-likelihood dN/dS from the 11 Bayes Empirical Bayes (BEB) classes were averaged separately among all ortholog and among all expansion fingers and the values were plotted in Figure 5. Among expansion fingers, the codeml assigned BEB classes appear to be skewed upwards because of the presence of many sites with high dN/dS and constraints in class assignment and curve fitting. An attempt to reduce this effect by using 21 BEB classes made little difference in the results. Thus, the expansion values (black bars) in Figure 5 are probably unrealistically high, especially among sites with a best dN/dS average below 1.0. To analyze zinc finger domain gain and loss, the same dataset was used as above. For each set of three orthologs, or for each paralog set from human and mouse, the number of intact canonical zinc finger repeats (C-X_2_-C-X_12_-H-X_3_-H) was counted in each protein.

### Zinc Finger Diversity and Logo Plots

To assess the amino acid diversity and relative entropy of amino acids in the C2H2 zinc fingers of poly-ZF proteins, all intact (X_7_-C-X_2_-C-X_12_-H-X_3_-H) zinc finger repeats were collected from each of the 12 main species analyzed. Zinc fingers containing a stop codon were removed, and alignment count, frequency and relative entropy plots for the 28-AA C2H2 zinc finger motif were generated using enoLOGOS [60]. In addition, amino acid diversity was explored by counting the frequency of each amino acid at positions −1, 3 and 6 of the α-helix among the set of zinc fingers used above, and by counting the frequency of each amino acid “triplet” of −1, 3 and 6.

## Supporting Information

Figure S1 ZF Proteins from Plants and Fungi. Poly-ZF proteins from several species of plants and fungi are shown. Zinc finger domains are boxed in red, linker sequences are underlined blue and the nucleotide-binding α-helix of each ZF domain is underlined green. The basic domain structure of tandem C2H2 zinc fingers separated by a 7-aa conserved linker is clearly present in plants and fungi, but the number of tandem zinc fingers is low and few poly-ZF genes are present in each species.
(0.79 MB TIF)

Figure S2 Cumulative BLAST Ratio of Poly-ZF Proteins. A cumulative BLAST ratio histogram for the poly-ZF gene families of four species analyzed. The BLAST ratio is the ratio of the blastp bit-score of the best hit to another protein from the same species to the bit-score of the identity match, and measures the similarity of a protein to its nearest paralog (see Methods). The cumulative histograms are a measure of the amount of recent duplication among poly-ZF genes in that species. *H. sapiens* poly-ZF proteins tend to be more closely related to their nearest relatives than those in *X. tropicalis*, followed by *C. intestinalis* and *C. elegans* poly-ZF proteins.
(0.14 MB TIF)

Figure S3 Human and Mouse Poly-ZF Subtree. A small section of the full neighbor-joining protein tree constructed with all mouse and human poly-ZF protein sequences. Leaves are Ensembl gene IDs, and the longest splice form of each gene was used to construct the protein tree. Human sequences are colored green and mouse sequences are colored blue. The tree is characterized by groups of one-to-one homology interspersed with species-specific gene expansions. Using our criterion of 5 sequences from one species with at most 1 sequence from the other, the clade defined by the red box and encompassing all sequences marked with a red circle was identified as a lineage-specific expansion and analyzed further. Ortholog pairs are marked with ‘**{**’ for comparison.
(0.51 MB TIF)

Figure S4 Position-Identity Correlation on Human Chr. 19. Histogram of the result of 25,000 trials of a random permutation test conducted on the poly-ZF proteins of human chromosome 19. In each case, Pearson's r is computed between genomic distance in bp and % amino acid identity. The correlation is −0.2 in the case of the real data, indicating that decreasing physical distance correlates with increasing AA identity.
(0.20 MB TIF)

Figure S5 Intragene Zinc Finger Alignments. Multiple alignments of the zinc fingers from each human KRAB-ZF protein with exactly 18 C2H2 zinc finger repeats. Below is a logo representation of amino acid diversity among 4,113 human zinc finger repeats shown for comparison. High levels of sequence diversity exist between the many fingers of each protein, and this diversity follows the same superficial pattern as diversity among fingers from many proteins. Arrows in the logo plot indicate positions −1, 3 and 6 respectively.
(20.72 MB TIF)

Figure S6 Linker Sequence Length Distribution. Distribution of the length of linker sequence between ZF repeats from all human and mouse poly-ZF gene family members. Each linker sequence was defined as the amino acid sequence situated between two adjacent C2H2 zinc fingers of canonical spacing. More than 86% of linker sequences have a length of 7 AA, representing the standard linker spacing for this gene family. Another peak at 35 AA is probably due to degraded ZF repeats that no longer match the canonical C2H2 ZF pattern (a 7 AA linker in addition to a 28 AA degraded zinc finger). Even among the human and mouse poly-ZF gene families, which contain proteins with many ZF repeats, most ZF repeats are organized in a tandem fashion including the very well conserved 7 amino-acid TGEKPYK linker sequence. This indicates that large, unbroken ZF arrays are a key feature of the gene family.
(0.25 MB TIF)

Table S1 Positive Selection in Human and Mouse Poly-ZF Expansions. Clades representing lineage-specific expansions in human and mouse poly-ZF proteins are shown. Listed for each clade are the species of origin, number of proteins represented, difference in likelihood between neutral and positive selection models, and p-value of evidence for positive selection. The p-value cutoff used was 0.05. Significance was measured using a likelihood ratio test (2*ΔLikelihood, compared to a χ^2^_2_; see Methods).
(0.05 MB DOC)

Table S2 Amino Acid Frequencies in ZF Domains. The amino acid frequencies at each of the three residues primarily responsible for DNA binding specificity among ZF domains. The frequency of each amino acid in the UniProt database (54.7) is also included for reference [62]. The amino acid distributions at each site are distinct from each other, and distinct from the universal protein average. Distributions were calculated over 23,797 C2H2 zinc finger domains collected from 12 species.
(0.06 MB DOC)

Dataset S1 Sequence sets used for analysis; ZF amino acid frequencies; Codeml results for poly-ZF expansions; Zinc Finger counts, orthologs and paralogs.
(3.77 MB ZIP)

## References

[1] Carroll SB, Grenier JK, Weatherbee SD (2005). From DNA to Diversity, 2nd edn.

[2] Lander ES, Linton LM, Birren B, Nusbaum C, Zody MC (2001). Initial sequencing and analysis of the human genome.. Nature.

[3] Venter JC, Adams MD, Myers EW, Li PW, Mural RJ (2001). The sequence of the human genome.. Science.

[4] Urrutia R (2003). KRAB-containing zinc-finger repressor proteins.. Genome Biol.

[5] Bellefroid EJ, Poncelet DA, Lecocq PJ, Revelant O, Martial JA (1991). The evolutionarily conserved Krüppel-associated box domain defines a subfamily of eukaryotic multifingered proteins.. Proc Natl Acad Sci U S A.

[6] Margolin JF, Friedman JR, Meyer WKH, Vissing H, Thiesen HJ (1994). Krüppel-associated boxes are potent transcriptional repression domains.. Proc Natl Acad Sci USA.

[7] Witzgall R, O'Leary E, Leaf A, Önaldi D, Bonventre JV (1994). The Krüppel-associated box-A (KRAB-A) domain of zinc finger proteins mediates transcriptional repression.. Proc Natl Acad Sci USA.

[8] Agata Y, Matsuda E, Shimizu A (1999). Two novel Krüppel-associated box-containing zinc-finger proteins, KRAZ1 and KRAZ2, repress transcription through functional interaction with the corepressor KAP-1 (TIF1beta/KRIP-1).. J Biol Chem.

[9] Gebelein B, Urrutia R (2001). Sequence-specific transcriptional repression by KS1, a multiple-zinc-finger-Krüppel-associated box protein.. Mol Cell Biol.

[10] Medugno L, Florio F, De Cegli R, Grosso M, Lupo A (2005). The Krüppel-like zinc-finger protein ZF224 represses aldolase A gene transcription by interacting with the KAP-1 co-repressor protein.. Gene.

[11] Peng H, Begg GE, Harper SL, Friedman JR, Speicher DW (2000). Biochemical analysis of the Krüppel-associated box (KRAB) transcriptional repression domain.. J Biol Chem.

[12] Ryan RF, Schultz DC, Ayyanathan K, Singh PB, Friedman JR (1999). KAP-1 corepressor protein interacts and colocalizes with heterochromatic and euchromatic HP1 proteins: a potential role for Krüppel-associated box-zinc finger proteins in heterochromatin-mediated gene silencing.. Mol Cell Biol.

[13] Friedman JR, Fredericks WJ, Jensen DE, Speicher DW, Huang XP (1996). KAP-1, a novel corepressor for the highly conserved KRAB repression domain.. Genes Dev.

[14] Underhill C, Qutob MS, Yee SP, Torchia J (2000). A Novel Nuclear Receptor Corepressor Complex, N-CoR, Contains Components of the Mammalian SWI/SNF Complex and the Corepressor KAP-1.. J Biol Chem.

[15] Schultz DC, Friedman JR, Rauscher FJ (2001). Targeting histone deacetylase complexes via KRAB-zinc finger proteins: the PHD and bromodomains of KAP-1 form a cooperative unit that recruits a novel isoform of the Mi-2α subunit of NuRD.. Genes Dev.

[16] Schultz DC, Ayyanathan K, Negorev D, Maul GG, Rauscher FJ (2002). SETDB1: a novel KAP-1-associated histone H3, lysine 9-specific methyltransferase that contributes to HP1-mediated silencing of euchromatic genes by KRAB zinc-finger proteins.. Genes Dev.

[17] Sripathy SP, Stevens J, Schultz DC (2006). The KAP1 Corepressor Functions to Coordinate the Assembly of De Novo HP1-Demarcated Microenvironments of Heterochromatin Required for KRAB Zinc Finger Protein-Mediated Transcriptional Repression.. Mol Cell Biol.

[18] Ayyanathan K, Lechner MS, Bell P, Maul GG, Schultz DC (2003). Regulated recruitment of HP1 to a euchromatic gene induces mitotically heritable, epigenetic gene silencing: a mammalian cell culture model of gene variegation.. Genes Dev.

[19] Wiznerowicz M, Jakobsson J, Szulc J, Liao S, Quazzola A (2007). The Krüppel-associated Box Repressor Domain Can Trigger de Novo Promoter Methylation during Mouse Early Embryogenesis.. J Biol Chem.

[20] Shoichet SA, Hoffmann K, Menzel C, Trautmann U, Moser B (2003). Mutations in the *ZNF41* gene are associated with cognitive deficits: identification of a new candidate for X-linked mental retardation.. Am J Hum Genet.

[21] Kleefstra T, Yntema HG, Oudakker AR, Banning MJ, Kalscheuer VM (2004). *Zinc finger 81* (*ZNF81*) mutations associated with X-linked mental retardation.. J Med Genet.

[22] Lugtenberg D, Yntema HG, Banning MJG, Oudakker AR, Firth HV (2005). *ZNF674*: A New Krüppel-Associated Box–Containing Zinc-Finger Gene Involved in Nonsyndromic X-Linked Mental Retardation.. Am J Hum Genet.

[23] Williams AJ, Khachigian LM, Shows T, Collins T (1995). Isolation and Characterization of a Novel Zinc-finger Protein with Transcriptional Repressor Activity.. J Biol Chem.

[24] Bardwell VJ, Treisman R (1994). The POZ domain: A conserved protein-protein interaction motif.. Genes Dev.

[25] Albagli O, Dhordain P, Deweindt C, Lecocq G, Leprince D (1995). The BTB/POZ Domain: A New Protein-Protein Interaction Motif Common to DNA- and Actin-binding Proteins.. Cell Growth Differ.

[26] Edelstein LC, Collins T (2005). The SCAN domain family of zinc finger transcription factors.. Gene.

[27] Choo Y, Klug A (1994). Selection of DNA binding sites for zinc fingers using rationally randomized DNA reveals coded interactions.. Proc Natl Acad Sci U S A.

[28] Iuchi S (2001). Three classes of C_2_H_2_ zinc finger proteins.. Cell Mol Life Sci.

[29] Birtle Z, Ponting CP (2006). Meisetz and the Birth of the KRAB Motif.. Bioinformatics.

[30] Lespinet O, Wolf YI, Koonin EV, Aravind L (2002). The Role of Lineage-Specific Gene Family Expansion in the Evolution of Eukaryotes.. Genome Res.

[31] Looman C, Abrink M, Mark C, Hellman L (2002). KRAB Zinc Finger Proteins: An Analysis of the Molecular Mechanisms Governing Their Increase in Numbers and Complexity During Evolution.. Mol Biol Evol.

[32] Hamilton AT, Huntley S, Tran-Gyamfi M, Baggott DM, Gordon L (2006). Evolutionary expansion and divergence in the ZF91 subfamily of primate-specific zinc finger genes.. Genome Res.

[33] Huntley S, Baggott DM, Hamilton AT, Tran-Gyamfi M, Yang S (2006). A comprehensive catalog of human KRAB-associated zinc finger genes: Insights into the evolutionary history of a large family of transcriptional repressors.. Genome Res.

[34] Bellefroid EJ, Marine JC, Tied T, Lecocq PJ, Rivière (1993). Clustered organization of homologous KRAB zinc-finger genes with enhanced expression in human T lymphoid cells.. EMBO J.

[35] Sander TL, Stringer KF, Maki JL, Szauter P, Stonr JR (2003). The SCAN domain defined a large family of zinc finger transcription factors.. Gene.

[36] Dehal P, Predki P, Olsen AS, Kobayashi A, Folta P (2001). Human Chromosome 19 and Related Regions in Mouse: Conservative and Lineage-Specific Evolution.. Science.

[37] Anisimova M, Bielawski JP, Yang Z (2001). Accuracy and power of the likelihood ratio test in detecting adaptive molecular evolution.. Mol Biol Evol.

[38] Wong WSW, Yang Z, Goldman N, Nielsen R (2004). Accuracy and power of Statistical Methods for Detecting Adaptive Evolution in Protein Coding Sequences and for Identifying Positively Selected Sites.. Genetics.

[39] Kim CA, Berg JM (1996). A 2.2 A resolution crystal structure of a designed zinc finger protein bound to DNA.. Nat Struct Biol.

[40] Elrod-Erickson M, Benson TE, Pabo CO (1998). High-resolution structures of variant Zif268-DNA complexes: implications for understanding zinc finger-DNA recognition.. Structure.

[41] Wuttke DS, Foster MP, Case DA, Gottesfeld JM, Wright PE (1997). Solution Structure of the First Three Zinc Fingers of TFIIIA Bound to the Cognate DNA Sequence: Determinants of Affinity and Sequence Specificity.. J Mol Biol.

[42] Yang Z (1997). PAML: a program package for phylogenetic analysis by maximum likelihood.. Computer Application in BioSciences.

[43] Yang Z, Wong WSW, Nielsen R (2005). Bayes Empirical Bayes Inference of Amino Acid Sites Under Positive Selection.. Mol Biol Evol.

[44] Shannon M, Hamilton AT, Gordon L, Branscomb E, Stubbs L (2003). Differential Expansion of Zinc-Finger Transcription Factor Loci in Homologous Human and Mouse Gene Clusters.. Genome Res.

[45] Schmidt D, Durrett R (2004). Adaptive Evolution Drives the Diversification of Zinc-Finger Binding Domains.. Mol Biol Evol.

[46] Filipova GN, Fagerlie S, Klenkova EM, Myers C, Dehner Y (1996). An Exceptionally Conserved Transcriptional Repressor, CTCF, Employs Different Combinations of Zinc Fingers To Bind Diverged Promoter Sequences of Avian and Mammalian c-myc Oncogenes.. Mol Cell Biol.

[47] Filipova GN, Qi C-F, Ulmer JE, Moore JM, Ward MD (2002). Tumor-associated Zinc Finger Mutation in the CTCF Transcription Factor Selectively Alter Its DNA-binding Specificity.. Cancer Res.

[48] Ishihara K, Oshimura M, Nakao M (2006). CTCF-Dependent Chromatin Insulator Is Linked to Epigenetic Remodeling.. Mol Cell.

[49] Wang ZX, Kueh JLL, Teh CHL, Rossbach M, Lim L (2007). Zfp206 Is A Transcription Factor That Controls Pluripotency of Embryonic Stem Cells.. Stem Cells.

[50] Krebs CJ, Larkins LK, Price R, Tullis KM, Miller RD (2003). Regulator of sex-limitation (Rsl) encodes a pair of KRAB zinc-finger genes that control sexually dimorphic liver gene expression.. Genes Dev.

[51] Horiba M, Martinez LD, Buescher JL, Sato S, Limoges J (2007). OTK18, a zinc-finger protein, regulates human immunodeficiency virus type 1 long terminal repeat through two distinct regulatory regions.. J Gen Virol.

[52] Garcia-Garcia MJ, Shibata M, Anderson KV (2008). Chato, a KRAB zinc-finger protein, regulates convergent extension in the mouse embryo.. Development.

[53] Pruitt KD, Tatusova T, Maglott DR (2007). NCBI reference sequences (RefSeq): a curated non-redundant sequence database of genomes, transcripts and proteins.. Nucleic Acids Res.

[54] Eddy SR (1998). Profile hidden Markov models.. Bioinformatics.

[55] Bateman A, Coin L, Durbin R, Finn RD, Hollich V (2004). The Pfam Protein Families Database:. Nucleic Acids Research.

[56] Cannon SB, Kozik A, Chan B, Michelmore R, Young ND (2003). DiagHunter and GenoPix2D: programs for genomic comparisons, large-scale homology discovery and visualization.. Genome Biology.

[57] Thompson JD, Higgins DG, Gibson TJ (1994). CLUSTAL W: improving the sensitivity of progressive multiple sequence alignment through sequence weighting, position-specific gap penalties and weight matrix choice.. Nucleic Acids Res.

[58] Sawyer SA (1989). Statistical tests for detecting gene conversion.. Mol Biol Evol.

[59] Crooks GE, Hon G, Chandonia JM, Brenner SE (2004). WebLogo: A sequence logo generator.. Genome Research.

[60] Workman CT, Yin Y, Corcoran DL, Ideker T, Stormo GD (2005). enoLOGOS: a versatile web tool for energy normalized sequence logos.. Nucleic Acids Research.

[61] Foster MP, Wuttke DS, Radhakrishnan I, Case DA, Gottesfeld JM (1997). Domain packing and dynamics in the DNA complex of the N-terminal zinc fingers of TFIIIA.. Nat Struct Biol.

[62] The UniProt Consortium (2008). The Universal Protein Resource (UniProt).. Nucleic Acids Res.

