# Supplementary figures and images for: Adaptive Evolution in Zinc Finger Transcription Factors
Source: PLoS Genet. 2009 Jan 2;5(1):e1000325. doi: 10.1371/journal.pgen.1000325 (PMC2604467; doi:10.1371/journal.pgen.1000325)

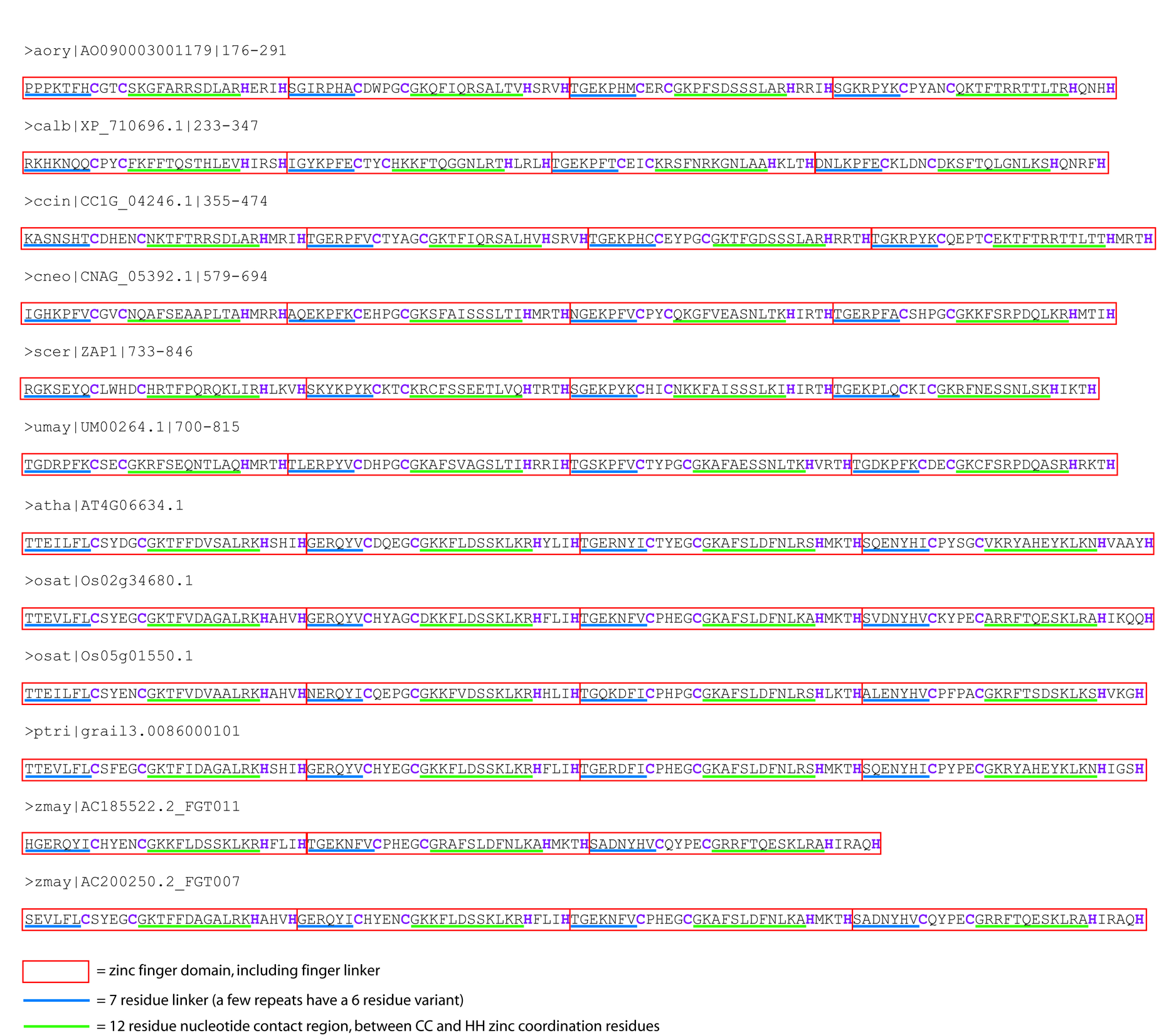

Supplement: Figure S1 — ZF Proteins from Plants and Fungi. Poly-ZF proteins from several species of plants and fungi are shown. Zinc finger domains are boxed in red, linker sequences are underlined blue and the nucleotide-binding α-helix of each ZF domain is underlined green. The basic domain structure of tandem C2H2 zinc fingers separated by a 7-aa conserved linker is clearly present in plants and fungi, but the number of tandem zinc fingers is low and few poly-ZF genes are present in each species. (0.79 MB TIF) [file pgen.1000325.s001.tif]

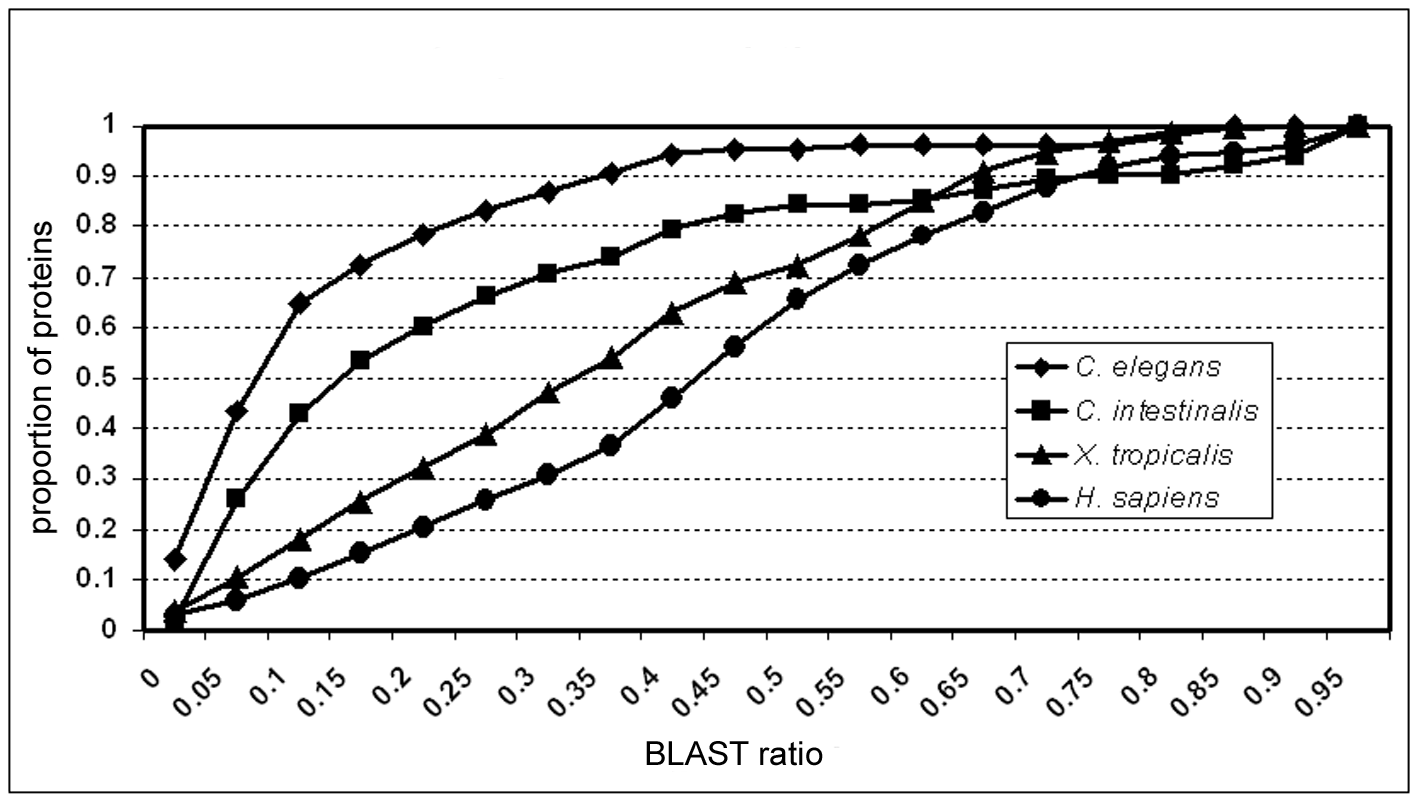

Supplement: Figure S2 — Cumulative BLAST Ratio of Poly-ZF Proteins. A cumulative BLAST ratio histogram for the poly-ZF gene families of four species analyzed. The BLAST ratio is the ratio of the blastp bit-score of the best hit to another protein from the same species to the bit-score of the identity match, and measures the similarity of a protein to its nearest paralog (see Methods). The cumulative histograms are a measure of the amount of recent duplication among poly-ZF genes in that species. H. sapiens poly-ZF proteins tend to be more closely related to their nearest relatives than those in X. tropicalis, followed by C. intestinalis and C. elegans poly-ZF proteins. (0.14 MB TIF) [file pgen.1000325.s002.tif]

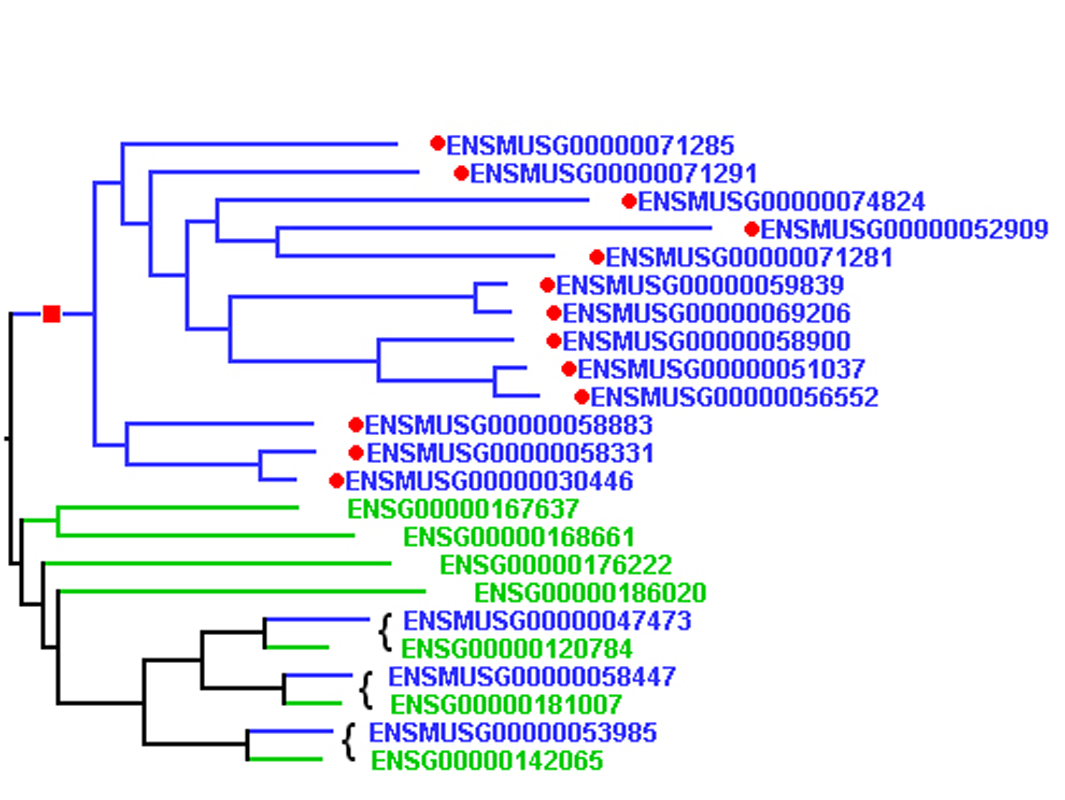

Supplement: Figure S3 — Human and Mouse Poly-ZF Subtree. A small section of the full neighbor-joining protein tree constructed with all mouse and human poly-ZF protein sequences. Leaves are Ensembl gene IDs, and the longest splice form of each gene was used to construct the protein tree. Human sequences are colored green and mouse sequences are colored blue. The tree is characterized by groups of one-to-one homology interspersed with species-specific gene expansions. Using our criterion of 5 sequences from one species with at most 1 sequence from the other, the clade defined by the red box and encompassing all sequences marked with a red circle was identified as a lineage-specific expansion and analyzed further. Ortholog pairs are marked with ‘{’ for comparison. (0.51 MB TIF) [file pgen.1000325.s003.tif]

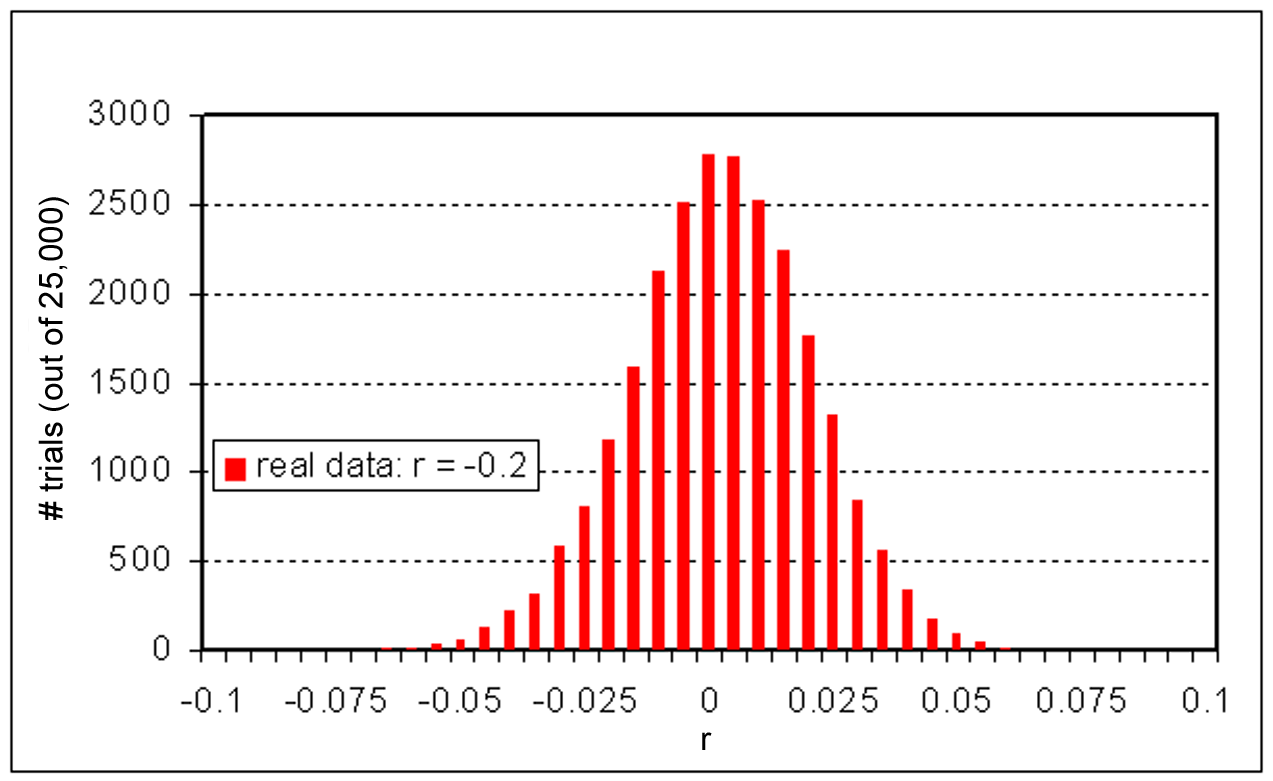

Supplement: Figure S4 — Position-Identity Correlation on Human Chr. 19. Histogram of the result of 25,000 trials of a random permutation test conducted on the poly-ZF proteins of human chromosome 19. In each case, Pearson's r is computed between genomic distance in bp and % amino acid identity. The correlation is −0.2 in the case of the real data, indicating that decreasing physical distance correlates with increasing AA identity. (0.20 MB TIF) [file pgen.1000325.s004.tif]

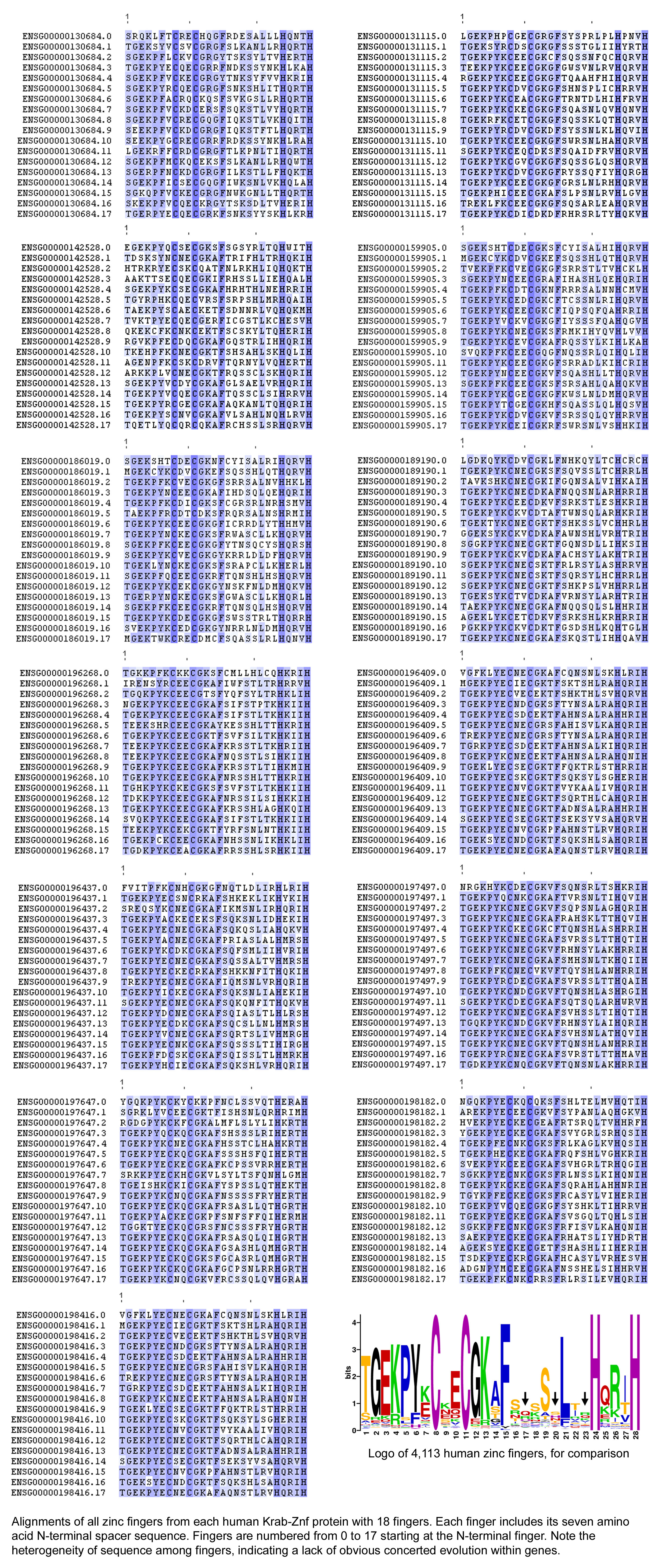

Supplement: Figure S5 — Intragene Zinc Finger Alignments. Multiple alignments of the zinc fingers from each human KRAB-ZF protein with exactly 18 C2H2 zinc finger repeats. Below is a logo representation of amino acid diversity among 4,113 human zinc finger repeats shown for comparison. High levels of sequence diversity exist between the many fingers of each protein, and this diversity follows the same superficial pattern as diversity among fingers from many proteins. Arrows in the logo plot indicate positions −1, 3 and 6 respectively. (20.72 MB TIF) [file pgen.1000325.s005.tif]

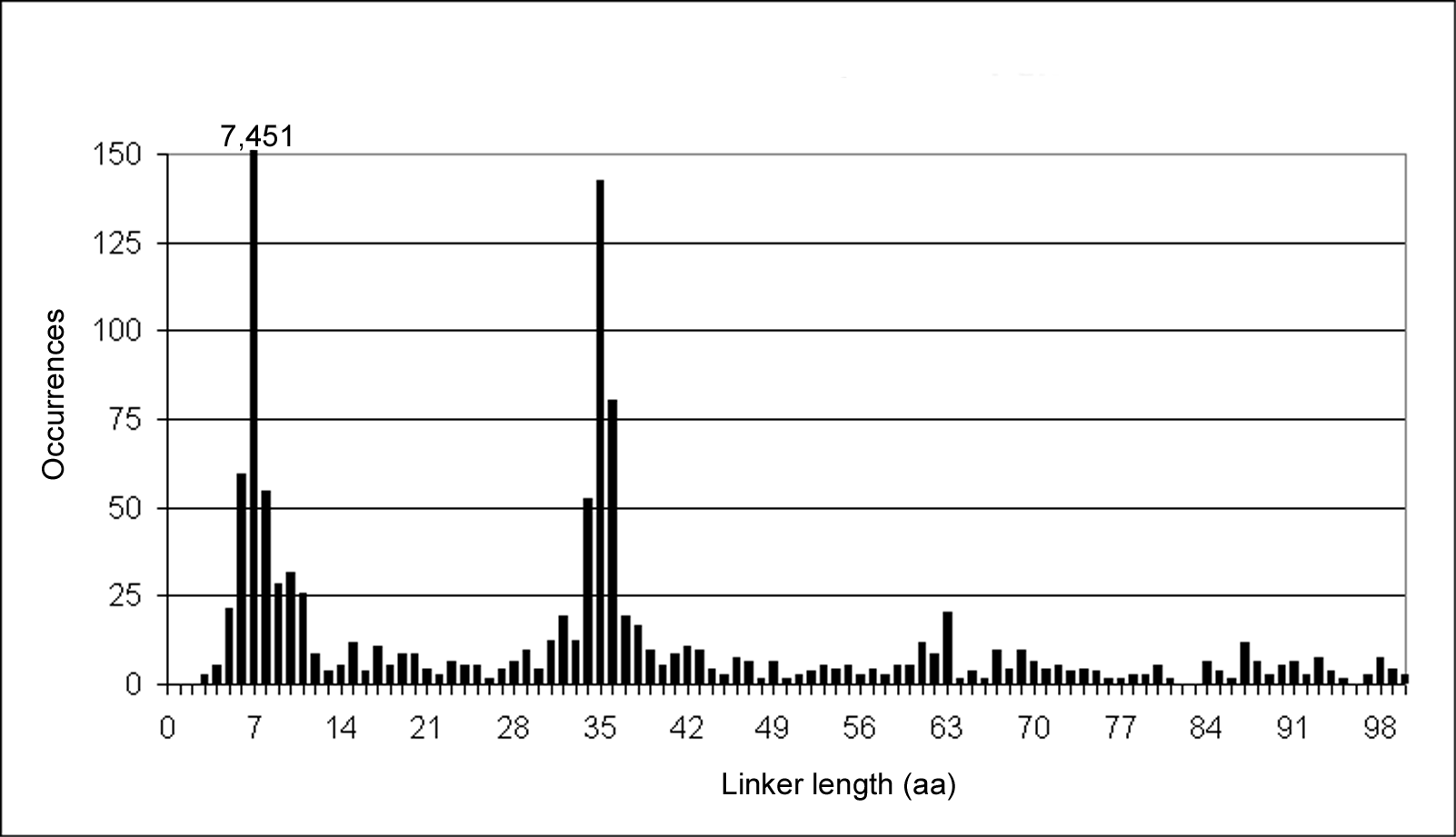

Supplement: Figure S6 — Linker Sequence Length Distribution. Distribution of the length of linker sequence between ZF repeats from all human and mouse poly-ZF gene family members. Each linker sequence was defined as the amino acid sequence situated between two adjacent C2H2 zinc fingers of canonical spacing. More than 86% of linker sequences have a length of 7 AA, representing the standard linker spacing for this gene family. Another peak at 35 AA is probably due to degraded ZF repeats that no longer match the canonical C2H2 ZF pattern (a 7 AA linker in addition to a 28 AA degraded zinc finger). Even among the human and mouse poly-ZF gene families, which contain proteins with many ZF repeats, most ZF repeats are organized in a tandem fashion including the very well conserved 7 amino-acid TGEKPYK linker sequence. This indicates that large, unbroken ZF arrays are a key feature of the gene family. (0.25 MB TIF) [file pgen.1000325.s006.tif]
