# Supplementary material for: Adaptive Evolution in Zinc Finger Transcription Factors
Source: PLoS Genet. 2009 Jan 2;5(1):e1000325. doi: 10.1371/journal.pgen.1000325 (PMC2604467; doi:10.1371/journal.pgen.1000325)
Supplement: Table S1 — Positive Selection in Human and Mouse Poly-ZF Expansions. Clades representing lineage-specific expansions in human and mouse poly-ZF proteins are shown. Listed for each clade are the species of origin, number of proteins represented, difference in likelihood between neutral and positive selection models, and p-value of evidence for positive selection. The p-value cutoff used was 0.05. Significance was measured using a likelihood ratio test (2*ΔLikelihood, compared to a χ2 2; see Methods). (0.05 MB DOC) [file pgen.1000325.s007.doc]

| **Species** | **# proteins** | **2ΔML** | **p-value** |
| --- | --- | --- | --- |
| Human | 9 | 28.7 | 1.4E-05 |
| Human | 5 | 33.2 | 1.5E-06 |
| Human | 5 | 0.7 | 1.7E+01 |
| Human | 7 | 2.3 | 7.7E+00 |
| Human | 5 | 10.5 | 1.3E-01 |
| Human | 5 | 8.3 | 3.8E-01 |
| Human | 8 | 18.4 | 2.4E-03 |
| Human | 17 | 21.5 | 5.2E-04 |
| Human | 5 | 25.1 | 8.5E-05 |
| Human | 10 | 6.1 | 1.2E+00 |
| Human | 7 | 4.2 | 3.0E+00 |
| Human | 33 | 381.6 | 3.4E-82 |
| Human | 18 | 102.8 | 1.1E-21 |
| Human | 5 | 60.5 | 1.7E-12 |
| Human | 6 | 13.3 | 3.1E-02 |
| Human | 5 | 18.5 | 2.3E-03 |
| Human | 7 | 4.4 | 2.7E+00 |
| Mouse | 5 | 11.1 | 9.2E-02 |
| Mouse | 19 | 21.3 | 5.7E-04 |
| Mouse | 11 | 8.1 | 4.2E-01 |
| Mouse | 9 | 6.6 | 8.9E-01 |
| Mouse | 6 | 13.7 | 2.6E-02 |
| Mouse | 22 | 112.9 | 7.5E-24 |
| Mouse | 13 | 49.5 | 4.2E-10 |
|  | | | |
| **Total** | **171/242 (71%) significant** | | |
