# Supplementary material for: Adaptive Evolution in Zinc Finger Transcription Factors
Source: PLoS Genet. 2009 Jan 2;5(1):e1000325. doi: 10.1371/journal.pgen.1000325 (PMC2604467; doi:10.1371/journal.pgen.1000325)
Supplement: Table S2 — Amino Acid Frequencies in ZF Domains. The amino acid frequencies at each of the three residues primarily responsible for DNA binding specificity among ZF domains. The frequency of each amino acid in the UniProt database (54.7) is also included for reference [62]. The amino acid distributions at each site are distinct from each other, and distinct from the universal protein average. Distributions were calculated over 23,797 C2H2 zinc finger domains collected from 12 species. (0.06 MB DOC) [file pgen.1000325.s008.doc]

| **Supplemental Table 2: AA frequencies in ZF domains** | | | | | | | | | | |
| --- | --- | --- | --- | --- | --- | --- | --- | --- | --- | --- |
| **Pos. -1:** | **Counts** | **Freq.** | **Pos. 3:** | **Counts** | **Freq.** | **Pos. 6:** | **Counts** | **Freq.** | **UniProt** | **Freq.** |
| **Q** | 4867 | 0.204522 | **S** | 3753 | 0.157709 | **R** | 4531 | 0.190402 | **L** | 0.0967 |
| **R** | 3840 | 0.161365 | **H** | 3495 | 0.146867 | **K** | 2706 | 0.113712 | **A** | 0.0804 |
| **S** | 2282 | 0.095894 | **N** | 3462 | 0.145481 | **Q** | 2610 | 0.109678 | **G** | 0.07 |
| **H** | 1731 | 0.07274 | **T** | 1719 | 0.072236 | **V** | 1943 | 0.081649 | **V** | 0.0679 |
| **T** | 1667 | 0.070051 | **A** | 1615 | 0.067866 | **I** | 1784 | 0.074967 | **S** | 0.0674 |
| **Y** | 1266 | 0.0532 | **D** | 1432 | 0.060176 | **T** | 1508 | 0.063369 | **E** | 0.067 |
| **D** | 1251 | 0.05257 | **Q** | 1357 | 0.057024 | **E** | 1474 | 0.061941 | **K** | 0.059 |
| **N** | 978 | 0.041098 | **Y** | 1156 | 0.048578 | **S** | 1323 | 0.055595 | **I** | 0.0589 |
| **K** | 916 | 0.038492 | **E** | 1054 | 0.044291 | **A** | 1214 | 0.051015 | **R** | 0.0548 |
| **L** | 894 | 0.037568 | **K** | 858 | 0.036055 | **L** | 1160 | 0.048746 | **D** | 0.0538 |
| **C** | 854 | 0.035887 | **G** | 845 | 0.035509 | **N** | 1051 | 0.044165 | **T** | 0.0537 |
| **E** | 636 | 0.026726 | **R** | 716 | 0.030088 | **Y** | 467 | 0.019624 | **P** | 0.0481 |
| **W** | 608 | 0.025549 | **L** | 708 | 0.029752 | **D** | 432 | 0.018154 | **N** | 0.0406 |
| **V** | 493 | 0.020717 | **V** | 523 | 0.021978 | **M** | 399 | 0.016767 | **Q** | 0.0395 |
| **F** | 424 | 0.017817 | **I** | 317 | 0.013321 | **G** | 341 | 0.01433 | **F** | 0.039 |
| **G** | 391 | 0.016431 | **F** | 236 | 0.009917 | **H** | 329 | 0.013825 | **Y** | 0.0296 |
| **A** | 273 | 0.011472 | **C** | 236 | 0.009917 | **C** | 310 | 0.013027 | **M** | 0.024 |
| **I** | 227 | 0.009539 | **M** | 207 | 0.008699 | **F** | 122 | 0.005127 | **H** | 0.0229 |
| **M** | 189 | 0.007942 | **P** | 86 | 0.003614 | **W** | 59 | 0.002479 | **C** | 0.0145 |
| **P** | 10 | 0.00042 | **W** | 22 | 0.000924 | **P** | 34 | 0.001429 | **W** | 0.011 |
|  | **23797** | **1** |  | **23797** | **1** |  | **23797** | **1** |  | **1** |
